# Supplementary material for: Quantitative Trait Loci and Candidate Genes That Control Seed Sugars Contents in the Soybean ‘Forrest’ by ‘Williams 82’ Recombinant Inbred Line Population
Source: Plants (Basel). 2023 Oct 8;12(19):3498. doi: 10.3390/plants12193498 (PMC10575016; doi:10.3390/plants12193498)
Supplement: Supplementary file 1 [file plants-12-03498-s001.zip › plants-2609617-supplementary.pdf]

Supplementary Results

Table S1. Quantitative trait loci (QTL) that control sugars (sucrose, stachyose, and raffinose) contents in FxW82 RIL population in Spring Lake, NC in 2018. These QTL have been identified by IM method.

Trait	QTL	Chr.	Marker/Interval	Position (cM)	LOD	R^2	Add. Eff.
Sucrose	*qSUC-1*	4	Gm04_7672403	6.5-8.5	4.89	11.33	1.358346
	qSUC-2	5	Gm05_3273418	35.01	2.07	9.13	-1.106173
	qSUC-3	6	Gm06_1737718-Gm06_5014399	46.7-52.4	10.10	21.87	-0.77401
	qSUC-4	10	Gm10_621706-Gm10_6020959	214.1-216.1	18.60	46.07	-5.38881
Stachyose	*qTAC-1*	2	Gm02_5142903-Gm02_5102501	133.2-133.4	2.22	5.23	0.056281
	qTAC-2	3	Gm03_4650811-Gm03_4699804	172.1-173.1	2.73	6.47	0.12403
	qTAC-3	5	Gm05_997108-Gm05_3290880	29.6-30.1	2.16	5.06	0.095345
Raffinose	*qRAF-1*	9	Gm09_4040711-Gm09_2821830	108.8-178.1	2.91	6.84	0.078535
	qRAF-2	11	Gm11_4796577-Gm11_2777893	0.1-0.2	2.13	5.06	0.095347
	qRAF-3	12	Gm12_6023395-Gm12_2379195	106.6-118.6	2.57	8.15	-0.018448
	qRAF-4	12	Gm12_553862-Gm12_1632399	177.3-183.3	2.32	5.99	0.01688
	qRAF-5	16	Gm16_390551-Gm16_2438982	0-0.1	2.14	5.08	0.094816
	qRAF-6	17	Gm17_3627810-Gm17_4789679	0-0.4	2.20	5.23	0.056278
	qRAF-7	18	Gm18_8937974-Gm18_2760770	96.2-96.5	2.91	6.84	0.078592
	qRAF-8	19	Gm19_3823921-Gm19_3424397	89.2-89.3	2.165	5.06	0.095346
	qRAF-9	19	Gm19_3789399-Gm19_4126523	98.1-128.7	3.467	8.13	-0.217482

Table S2. Quantitative trait loci (QTL) that control sugars (sucrose, stachyose, and raffinose) contents in FxW82 RIL population in Carbondale, IL in 2020. These QTL have been identified by IM method.

Trait	QTL	Chr.	Marker/Interval	Position (cM)	LOD	R^2	Add. Eff.
Sucrose	*qSUC-1*	2	Gm02_1199805-Gm02_1207820	196.4-197.3	2.81	4.31	-0.173664
	qSUC-2	5	Gm05_3803682-Gm05_3726014	18.1-22.6	2.27	3.5	-0.155954
	qSUC-3	8	Gm08_5960619-Gm08_8268861	43.1-51.1	2.51	4.95	0.1854
Stachyose	*qSTA-1*	3	Gm03_4072778	42.21	2.18	14.45	0.266102
	qSTA-2	20	Gm20_51113	146.51	2.25	3.46	-0.104184
Raffinose	*qRAF-1*	1	Gm01_3466825	4.11	127.70	88.85	1.897774

Table S3. Comparison of the Williams 82 and Forrest cv. Sequences of the Glyma.09G073600, Glyma.08G143500, Glyma.17G111400, Glyma.17G035800, Glyma.09G016600 and Glyma.05G003900 candidate genes.

Gene ID	Position	Region	Williams 82	Forrest	Amino Acid Changes
**Glyma.09G073600**	7810150	3'UTR	-	AAA	
	7810151	3'UTR	T	A	
	7810586	3'UTR	C	A	
	7812087	Intron	G	A	
	7812135	Exon 11	A	G	I > I
	7812192	Exon 11	A	G	G > G
	7812358	Intron	T	G	
	7812662	Intron	T	A	
	7812663	Intron	G	A	
	7812808	Exon 9	A	C	L > L

	7812878- 7812924	Intron	AAATGCTTGAACCGTG TCAAAGTTTTGAGTAA AAGCTAAATTTCACT	-	
	7813121	Exon 8	T	C	A > A
	7813201	Intron	C	T	
	7813238	Intron	A	T	
	7813408	Intron	A	T	
	7813438	Intron	C	T	
	7813593	Exon 6	T	C	A > A
	7813596	Exon 6	C	T	E > E
	7813689	Exon 6	C	T	E > E
	7813861- 7813860	Intron	-	A	
	7813926	Intron	A	G	
	7813954	Intron	T	A	
	7814003	Intron	C	A	
	7814015	Intron	T	A	
	7814072	Intron	C	T	
	7814152	Intron	A	G	
	7814164- 7814163	Intron	-	AG	
	7814383- 7814386	Intron	AAAA	-	
	7814423	Intron	G	A	
	7814501	Intron	T	C	
	7815161-7815160	Intron	-	TTTTTTC	
	7815461	5'UTR	G	A	
	7815714	5'UTR	T	A	
	7816084- 7816083	Intron	-	AA	
	7816212	5'UTR	T	G	
**Glyma.08G143500**	10949852- 10949862	3' UTR	TATCCCTGTCG	-	
	10950188	Exon 7	A	G	L > S
	10950511	Intron	G	C	
	10951908	Intron	T	C	
	10952318	Intron	A	G	
	10952623	Intron	C	T	
	10952653	Intron	G	A	
	10952746	Intron	T	C	
	10952778	Intron	A	G	
	10952778	Intron	A	G	
	10952981	Intron	C	T	
	10953210	Intron	C	T	
	10953227	Intron	G	A	
	10953363	Intron	A	G	
	10953405- 10953410	Intron	CCCCCC	-	
	10954104	Intron	G	T	
	10954380	Intron	G	A	
	10954380	Intron	G	A	
	10954692	Intron	A	T	
	10954836 - 10954835	Intron	-	CCAAGATT	
	10955679 - 10955679	Intron	A	-	
	10955856 - 10955858	Intron	AAA	-	
	10955892	Intron	C	G	
	10956059	5'UTR	G	T	

	10956208	5'UTR	T	A	
**Glyma.17G111400**	8744745	5'UTR	T	C	
	8745755	Exon 3	G	A	Q > Q
	8745761	Exon 3	C	T	L > L
	8745794	Exon 3	C	A	I > I
	8745950	Exon 3	A	G	S > S
	8746028	Exon 3	G	A	E > E
	8746454	Exon 3	G	A	A > A
	8,747,340 - 8,747,342	3'UTR	GTT	-	
**Glyma.17G035800**	2630991	Intron	C	T	
	2637731	Intron	G	A	
**Glyma.09G016600**	1285133	Exon 1	A	G	M > V
	1285854	Intron	C	A	
	1286228	Intron	C	T	
	1286241	Intron	C	T	
	1,286,529 - 1,286,528	Exon 2	-	ATA	Frameshift
	1286875	Intron	T	C	
	1287390	Intron	T	C	
	1288323	Intron	T	C	
	1,288,679 - 1,288,688	Exon 8	CTCACTAGAC	-	Frameshift
	1288886	Intron	A	G	
	1289544	Intron	T	G	
	1290157	Exon 12	C	T	S > F
	1290346	Intron	A	C	
	1290432	Intron	A	C	
**Glyma.05G003900**	307548	Exon 1	C	G	P > A
	308102	Exon 1	C	A	T > T
	308379	Intron	C	A	
	308412	Intron	G	A	
	308749	Exon 2	C	T	Y > Y
	308753	Exon 2	C	G	R > G
	308885	Intron	A	T	
	309282	Intron	C	T	
	309407	Intron	C	A	
	309422	Intron	T	-	

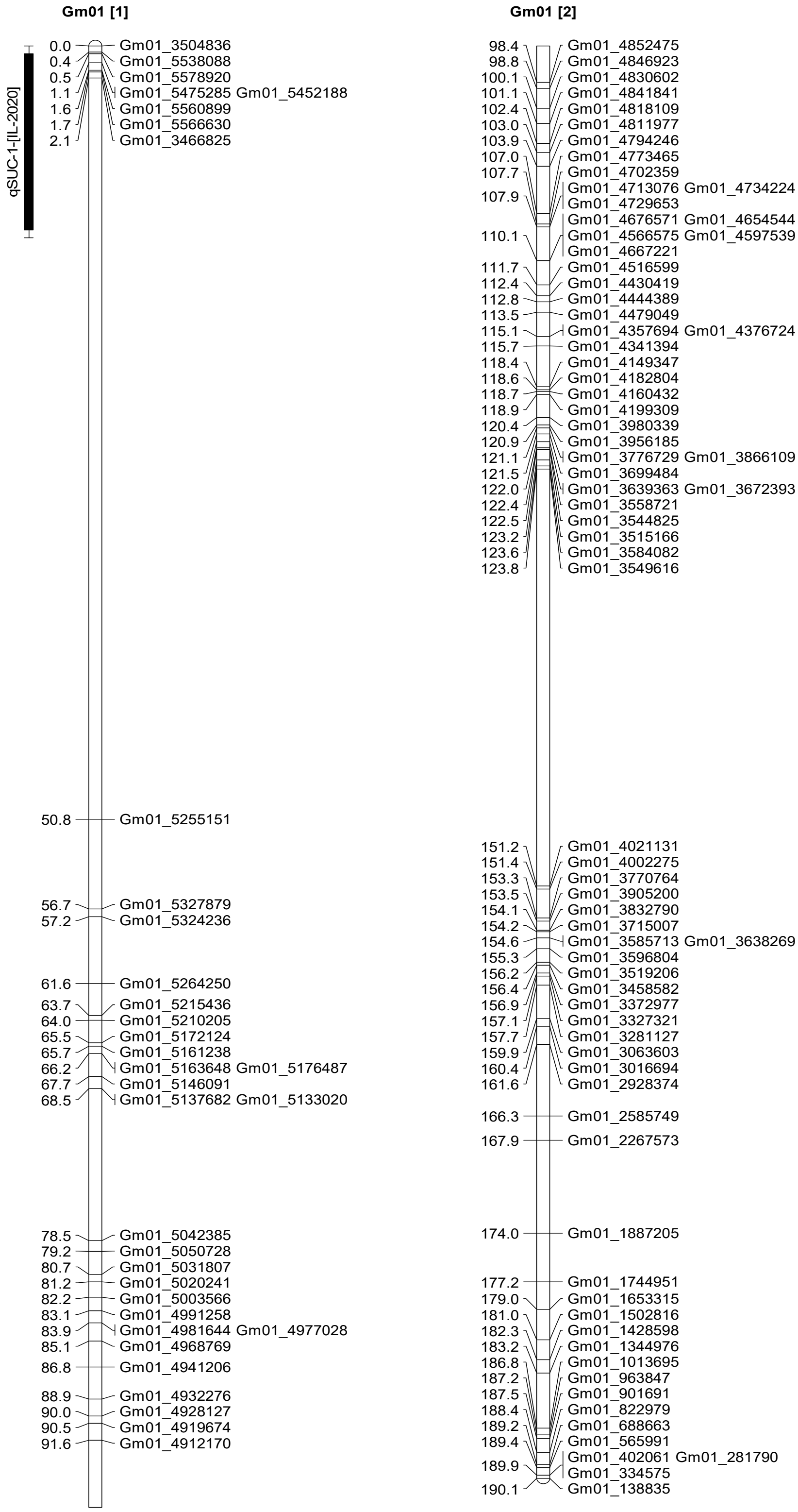
Gm01 [1]
Gm01 [2]
qSUC-1-[IL-2020]
0.0 Gm01_3504836
0.4 Gm01_5538088
0.5 Gm01_5578920
1.1 Gm01_5475285 Gm01_5452188
1.6 Gm01_5560899
1.7 Gm01_5566630
2.1 Gm01_3466825
50.8 Gm01_5255151
56.7 Gm01_5327879
57.2 Gm01_5324236
61.6 Gm01_5264250
63.7 Gm01_5215436
64.0 Gm01_5210205
65.5 Gm01_5172124
65.7 Gm01_5161238
66.2 Gm01_5163648 Gm01_5176487
67.7 Gm01_5146091
68.5 Gm01_5137682 Gm01_5133020
78.5 Gm01_5042385
79.2 Gm01_5050728
80.7 Gm01_5031807
81.2 Gm01_5020241
82.2 Gm01_5003566
83.1 Gm01_4991258
83.9 Gm01_4981644 Gm01_4977028
85.1 Gm01_4968769
86.8 Gm01_4941206
88.9 Gm01_4932276
90.0 Gm01_4928127
90.5 Gm01_4919674
91.6 Gm01_4912170
98.4 Gm01_4852475
98.8 Gm01_4846923
100.1 Gm01_4830602
101.1 Gm01_4841841
102.4 Gm01_4818109
103.0 Gm01_4811977
103.9 Gm01_4794246
107.0 Gm01_4773465
107.7 Gm01_4702359
107.9 Gm01_4713076 Gm01_4734224
Gm01_4729653
110.1 Gm01_4676571 Gm01_4654544
Gm01_4566575 Gm01_4597539
Gm01_4667221
111.7 Gm01_4516599
112.4 Gm01_4430419
112.8 Gm01_4444389
113.5 Gm01_4479049
115.1 Gm01_4357694 Gm01_4376724
115.7 Gm01_4341394
118.4 Gm01_4149347
118.6 Gm01_4182804
118.7 Gm01_4160432
118.9 Gm01_4199309
120.4 Gm01_3980339
120.9 Gm01_3956185
121.1 Gm01_3776729 Gm01_3866109
121.5 Gm01_3699484
122.0 Gm01_3639363 Gm01_3672393
122.4 Gm01_3558721
122.5 Gm01_3544825
123.2 Gm01_3515166
123.6 Gm01_3584082
123.8 Gm01_3549616
151.2 Gm01_4021131
151.4 Gm01_4002275
153.3 Gm01_3770764
153.5 Gm01_3905200
154.1 Gm01_3832790
154.2 Gm01_3715007
154.6 Gm01_3585713 Gm01_3638269
155.3 Gm01_3596804
156.2 Gm01_3519206
156.4 Gm01_3458582
156.9 Gm01_3372977
157.1 Gm01_3327321
157.7 Gm01_3281127
159.9 Gm01_3063603
160.4 Gm01_3016694
161.6 Gm01_2928374
166.3 Gm01_2585749
167.9 Gm01_2267573
174.0 Gm01_1887205
177.2 Gm01_1744951
179.0 Gm01_1653315
181.0 Gm01_1502816
182.3 Gm01_1428598
183.2 Gm01_1344976
186.8 Gm01_1013695
187.2 Gm01_963847
187.5 Gm01_901691
188.4 Gm01_822979
189.2 Gm01_688663
189.4 Gm01_565991
189.9 Gm01_402061 Gm01_281790
Gm01_334575
190.1 Gm01_138835

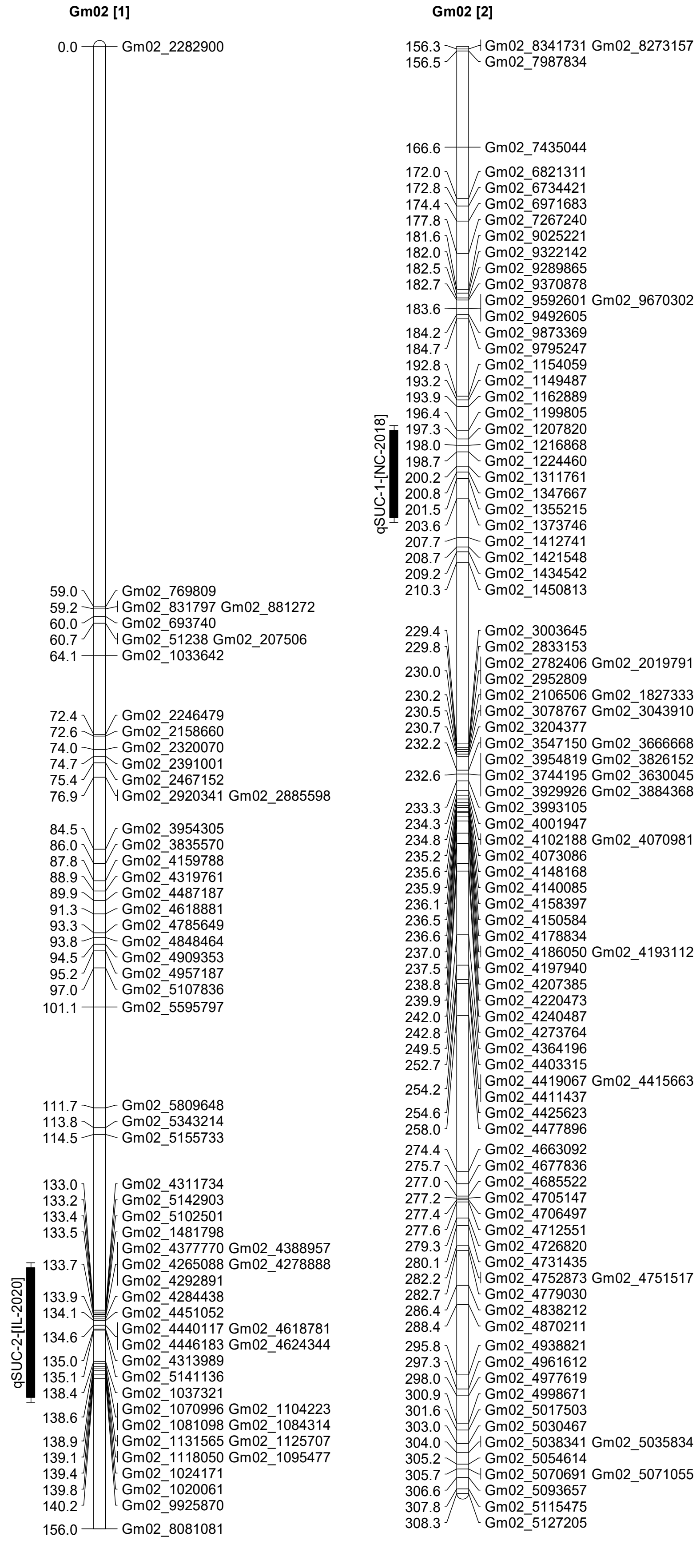

Gm02 [1]
0.0 Gm02_2282900
59.0 Gm02_769809
59.2 Gm02_831797 Gm02_881272
60.0 Gm02_693740
60.7 Gm02_51238 Gm02_207506
64.1 Gm02_1033642
72.4 Gm02_2246479
72.6 Gm02_2158660
74.0 Gm02_2320070
74.7 Gm02_2391001
75.4 Gm02_2467152
76.9 Gm02_2920341 Gm02_2885598
84.5 Gm02_3954305
86.0 Gm02_3835570
87.8 Gm02_4159788
88.9 Gm02_4319761
89.9 Gm02_4487187
91.3 Gm02_4618881
93.3 Gm02_4785649
93.8 Gm02_4848464
94.5 Gm02_4909353
95.2 Gm02_4957187
97.0 Gm02_5107836
101.1 Gm02_5595797
111.7 Gm02_5809648
113.8 Gm02_5343214
114.5 Gm02_5155733
133.0 Gm02_4311734
133.2 Gm02_5142903
133.4 Gm02_5102501
133.5 Gm02_1481798
Gm02_4377770 Gm02_4388957
133.7 Gm02_4265088 Gm02_4278888
Gm02_4292891
133.9 Gm02_4284438
134.1 Gm02_4451052
134.6 Gm02_4440117 Gm02_4618781
Gm02_4446183 Gm02_4624344
135.0 Gm02_4313989
135.1 Gm02_5141136
138.4 Gm02_1037321
138.6 Gm02_1070996 Gm02_1104223
Gm02_1081098 Gm02_1084314
138.9 Gm02_1131565 Gm02_1125707
139.1 Gm02_1118050 Gm02_1095477
139.4 Gm02_1024171
139.8 Gm02_1020061
140.2 Gm02_9925870
156.0 Gm02_8081081
qSUC-2-[IL-2020]
Gm02 [2]
156.3 Gm02_8341731 Gm02_8273157
156.5 Gm02_7987834
166.6 Gm02_7435044
172.0 Gm02_6821311
172.8 Gm02_6734421
174.4 Gm02_6971683
177.8 Gm02_7267240
181.6 Gm02_9025221
182.0 Gm02_9322142
182.5 Gm02_9289865
182.7 Gm02_9370878
183.6 Gm02_9592601 Gm02_9670302
Gm02_9492605
184.2 Gm02_9873369
184.7 Gm02_9795247
192.8 Gm02_1154059
193.2 Gm02_1149487
193.9 Gm02_1162889
196.4 Gm02_1199805
197.3 Gm02_1207820
198.0 Gm02_1216868
198.7 Gm02_1224460
200.2 Gm02_1311761
200.8 Gm02_1347667
201.5 Gm02_1355215
203.6 Gm02_1373746
207.7 Gm02_1412741
208.7 Gm02_1421548
209.2 Gm02_1434542
210.3 Gm02_1450813
qSUC-1-[NC-2018]
229.4 Gm02_3003645
229.8 Gm02_2833153
230.0 Gm02_2782406 Gm02_2019791
Gm02_2952809
230.2 Gm02_2106506 Gm02_1827333
230.5 Gm02_3078767 Gm02_3043910
230.7 Gm02_3204377
232.2 Gm02_3547150 Gm02_3666668
Gm02_3954819 Gm02_3826152
232.6 Gm02_3744195 Gm02_3630045
Gm02_3929926 Gm02_3884368
233.3 Gm02_3993105
234.3 Gm02_4001947
234.8 Gm02_4102188 Gm02_4070981
235.2 Gm02_4073086
235.6 Gm02_4148168
235.9 Gm02_4140085
236.1 Gm02_4158397
236.5 Gm02_4150584
236.6 Gm02_4178834
237.0 Gm02_4186050 Gm02_4193112
237.5 Gm02_4197940
238.8 Gm02_4207385
239.9 Gm02_4220473
242.0 Gm02_4240487
242.8 Gm02_4273764
249.5 Gm02_4364196
252.7 Gm02_4403315
254.2 Gm02_4419067 Gm02_4415663
Gm02_4411437
254.6 Gm02_4425623
258.0 Gm02_4477896
274.4 Gm02_4663092
275.7 Gm02_4677836
277.0 Gm02_4685522
277.2 Gm02_4705147
277.4 Gm02_4706497
277.6 Gm02_4712551
279.3 Gm02_4726820
280.1 Gm02_4731435
282.2 Gm02_4752873 Gm02_4751517
282.7 Gm02_4779030
286.4 Gm02_4838212
288.4 Gm02_4870211
295.8 Gm02_4938821
297.3 Gm02_4961612
298.0 Gm02_4977619
300.9 Gm02_4998671
301.6 Gm02_5017503
303.0 Gm02_5030467
304.0 Gm02_5038341 Gm02_5035834
305.2 Gm02_5054614
305.7 Gm02_5070691 Gm02_5071055
306.6 Gm02_5093657
307.8 Gm02_5115475
308.3 Gm02_5127205

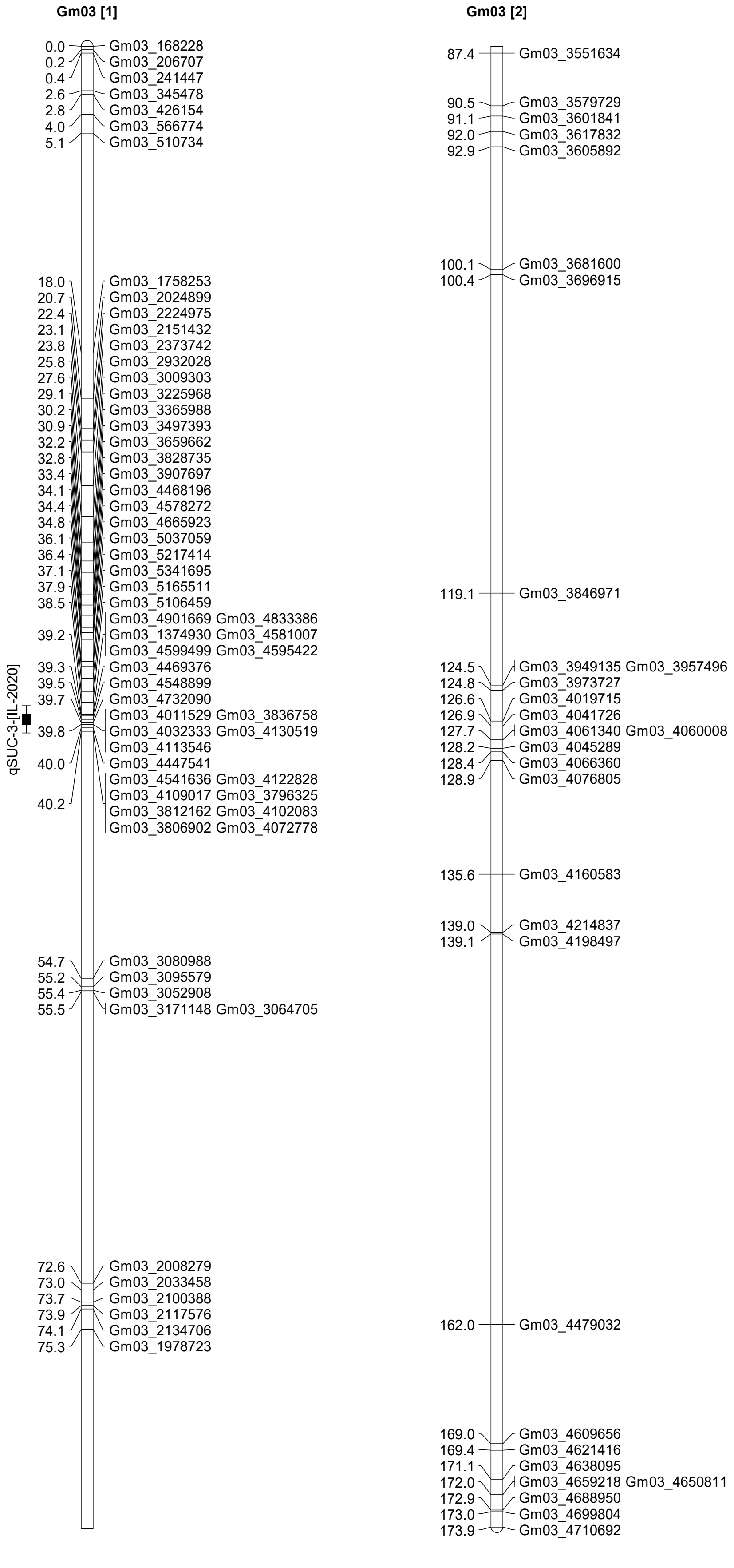

Gm03 [1]
0.0 Gm03_168228
0.2 Gm03_206707
0.4 Gm03_241447
2.6 Gm03_345478
2.8 Gm03_426154
4.0 Gm03_566774
5.1 Gm03_510734
18.0 Gm03_1758253
20.7 Gm03_2024899
22.4 Gm03_2224975
23.1 Gm03_2151432
23.8 Gm03_2373742
25.8 Gm03_2932028
27.6 Gm03_3009303
29.1 Gm03_3225968
30.2 Gm03_3365988
30.9 Gm03_3497393
32.2 Gm03_3659662
32.8 Gm03_3828735
33.4 Gm03_3907697
34.1 Gm03_4468196
34.4 Gm03_4578272
34.8 Gm03_4665923
36.1 Gm03_5037059
36.4 Gm03_5217414
37.1 Gm03_5341695
37.9 Gm03_5165511
38.5 Gm03_5106459
39.2 Gm03_4901669 Gm03_4833386
Gm03_1374930 Gm03_4581007
Gm03_4599499 Gm03_4595422
39.3 Gm03_4469376
39.5 Gm03_4548899
39.7 Gm03_4732090
39.8 Gm03_4011529 Gm03_3836758
Gm03_4032333 Gm03_4130519
Gm03_4113546
40.0 Gm03_4447541
40.2 Gm03_4541636 Gm03_4122828
Gm03_4109017 Gm03_3796325
Gm03_3812162 Gm03_4102083
Gm03_3806902 Gm03_4072778
qSUC-3-[IL-2020]
54.7 Gm03_3080988
55.2 Gm03_3095579
55.4 Gm03_3052908
55.5 Gm03_3171148 Gm03_3064705
72.6 Gm03_2008279
73.0 Gm03_2033458
73.7 Gm03_2100388
73.9 Gm03_2117576
74.1 Gm03_2134706
75.3 Gm03_1978723
Gm03 [2]
87.4 Gm03_3551634
90.5 Gm03_3579729
91.1 Gm03_3601841
92.0 Gm03_3617832
92.9 Gm03_3605892
100.1 Gm03_3681600
100.4 Gm03_3696915
119.1 Gm03_3846971
124.5 Gm03_3949135 Gm03_3957496
124.8 Gm03_3973727
126.6 Gm03_4019715
126.9 Gm03_4041726
127.7 Gm03_4061340 Gm03_4060008
128.2 Gm03_4045289
128.4 Gm03_4066360
128.9 Gm03_4076805
135.6 Gm03_4160583
139.0 Gm03_4214837
139.1 Gm03_4198497
162.0 Gm03_4479032
169.0 Gm03_4609656
169.4 Gm03_4621416
171.1 Gm03_4638095
172.0 Gm03_4659218 Gm03_4650811
172.9 Gm03_4688950
173.0 Gm03_4699804
173.9 Gm03_4710692

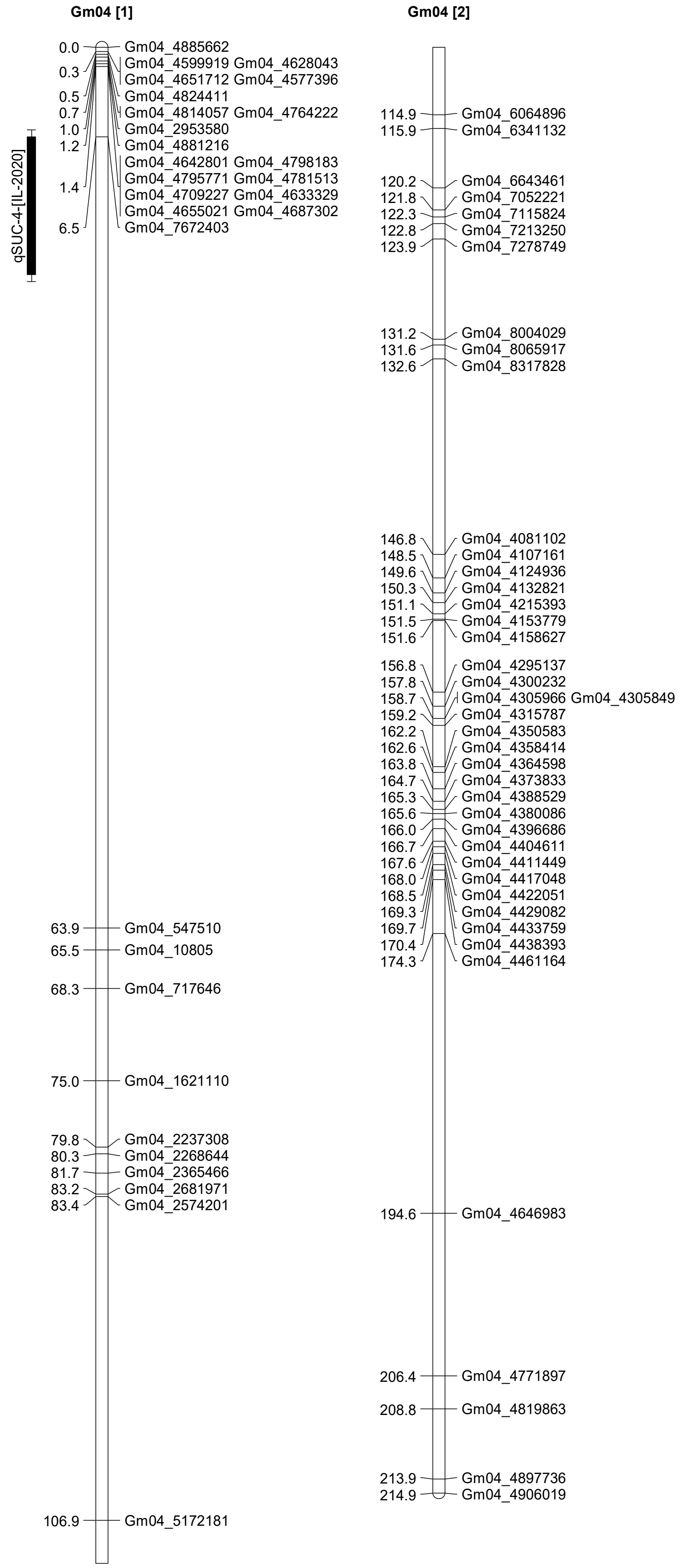
Gm04 [1]
Gm04 [2]
0.0 Gm04_4885662
0.3 Gm04_4599919 Gm04_4628043
Gm04_4651712 Gm04_4577396
0.5 Gm04_4824411
0.7 Gm04_4814057 Gm04_4764222
1.0 Gm04_2953580
1.2 Gm04_4881216
Gm04_4642801 Gm04_4798183
1.4 Gm04_4795771 Gm04_4781513
Gm04_4709227 Gm04_4633329
Gm04_4655021 Gm04_4687302
6.5 Gm04_7672403
qSUC-4-[IL-2020]
63.9 Gm04_547510
65.5 Gm04_10805
68.3 Gm04_717646
75.0 Gm04_1621110
79.8 Gm04_2237308
80.3 Gm04_2268644
81.7 Gm04_2365466
83.2 Gm04_2681971
83.4 Gm04_2574201
106.9 Gm04_5172181
114.9 Gm04_6064896
115.9 Gm04_6341132
120.2 Gm04_6643461
121.8 Gm04_7052221
122.3 Gm04_7115824
122.8 Gm04_7213250
123.9 Gm04_7278749
131.2 Gm04_8004029
131.6 Gm04_8065917
132.6 Gm04_8317828
146.8 Gm04_4081102
148.5 Gm04_4107161
149.6 Gm04_4124936
150.3 Gm04_4132821
151.1 Gm04_4215393
151.5 Gm04_4153779
151.6 Gm04_4158627
156.8 Gm04_4295137
157.8 Gm04_4300232
158.7 Gm04_4305966 Gm04_4305849
159.2 Gm04_4315787
162.2 Gm04_4350583
162.6 Gm04_4358414
163.8 Gm04_4364598
164.7 Gm04_4373833
165.3 Gm04_4388529
165.6 Gm04_4380086
166.0 Gm04_4396686
166.7 Gm04_4404611
167.6 Gm04_4411449
168.0 Gm04_4417048
168.5 Gm04_4422051
169.3 Gm04_4429082
169.7 Gm04_4433759
170.4 Gm04_4438393
174.3 Gm04_4461164
194.6 Gm04_4646983
206.4 Gm04_4771897
208.8 Gm04_4819863
213.9 Gm04_4897736
214.9 Gm04_4906019

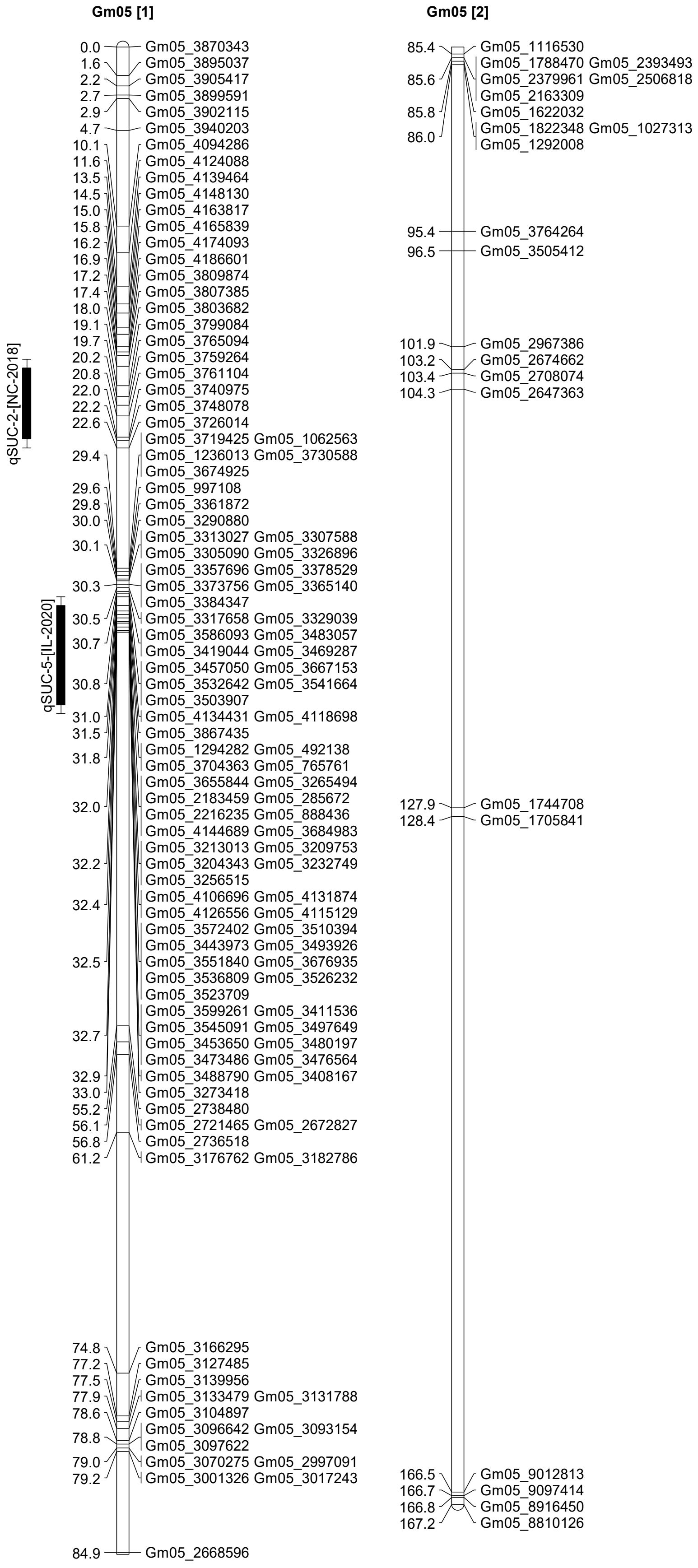
Gm05 [1]
Gm05 [2]
qSUC-2-[NC-2018]
qSUC-5-[IL-2020]
0.0 Gm05_3870343
1.6 Gm05_3895037
2.2 Gm05_3905417
2.7 Gm05_3899591
2.9 Gm05_3902115
4.7 Gm05_3940203
10.1 Gm05_4094286
11.6 Gm05_4124088
13.5 Gm05_4139464
14.5 Gm05_4148130
15.0 Gm05_4163817
15.8 Gm05_4165839
16.2 Gm05_4174093
16.9 Gm05_4186601
17.2 Gm05_3809874
17.4 Gm05_3807385
18.0 Gm05_3803682
19.1 Gm05_3799084
19.7 Gm05_3765094
20.2 Gm05_3759264
20.8 Gm05_3761104
22.0 Gm05_3740975
22.2 Gm05_3748078
22.6 Gm05_3726014
Gm05_3719425 Gm05_1062563
29.4 Gm05_1236013 Gm05_3730588
Gm05_3674925
29.6 Gm05_997108
29.8 Gm05_3361872
30.0 Gm05_3290880
Gm05_3313027 Gm05_3307588
30.1 Gm05_3305090 Gm05_3326896
Gm05_3357696 Gm05_3378529
30.3 Gm05_3373756 Gm05_3365140
Gm05_3384347
30.5 Gm05_3317658 Gm05_3329039
Gm05_3586093 Gm05_3483057
30.7 Gm05_3419044 Gm05_3469287
Gm05_3457050 Gm05_3667153
30.8 Gm05_3532642 Gm05_3541664
Gm05_3503907
31.0 Gm05_4134431 Gm05_4118698
31.5 Gm05_3867435
Gm05_1294282 Gm05_492138
31.8 Gm05_3704363 Gm05_765761
Gm05_3655844 Gm05_3265494
Gm05_2183459 Gm05_285672
32.0 Gm05_2216235 Gm05_888436
Gm05_4144689 Gm05_3684983
Gm05_3213013 Gm05_3209753
32.2 Gm05_3204343 Gm05_3232749
Gm05_3256515
Gm05_4106696 Gm05_4131874
32.4 Gm05_4126556 Gm05_4115129
Gm05_3572402 Gm05_3510394
Gm05_3443973 Gm05_3493926
32.5 Gm05_3551840 Gm05_3676935
Gm05_3536809 Gm05_3526232
Gm05_3523709
Gm05_3599261 Gm05_3411536
Gm05_3545091 Gm05_3497649
32.7 Gm05_3453650 Gm05_3480197
Gm05_3473486 Gm05_3476564
32.9 Gm05_3488790 Gm05_3408167
33.0 Gm05_3273418
55.2 Gm05_2738480
56.1 Gm05_2721465 Gm05_2672827
56.8 Gm05_2736518
61.2 Gm05_3176762 Gm05_3182786
74.8 Gm05_3166295
77.2 Gm05_3127485
77.5 Gm05_3139956
77.9 Gm05_3133479 Gm05_3131788
78.6 Gm05_3104897
Gm05_3096642 Gm05_3093154
78.8 Gm05_3097622
79.0 Gm05_3070275 Gm05_2997091
79.2 Gm05_3001326 Gm05_3017243
84.9 Gm05_2668596
85.4 Gm05_1116530
Gm05_1788470 Gm05_2393493
85.6 Gm05_2379961 Gm05_2506818
Gm05_2163309
85.8 Gm05_1622032
Gm05_1822348 Gm05_1027313
86.0 Gm05_1292008
95.4 Gm05_3764264
96.5 Gm05_3505412
101.9 Gm05_2967386
103.2 Gm05_2674662
103.4 Gm05_2708074
104.3 Gm05_2647363
127.9 Gm05_1744708
128.4 Gm05_1705841
166.5 Gm05_9012813
166.7 Gm05_9097414
166.8 Gm05_8916450
167.2 Gm05_8810126

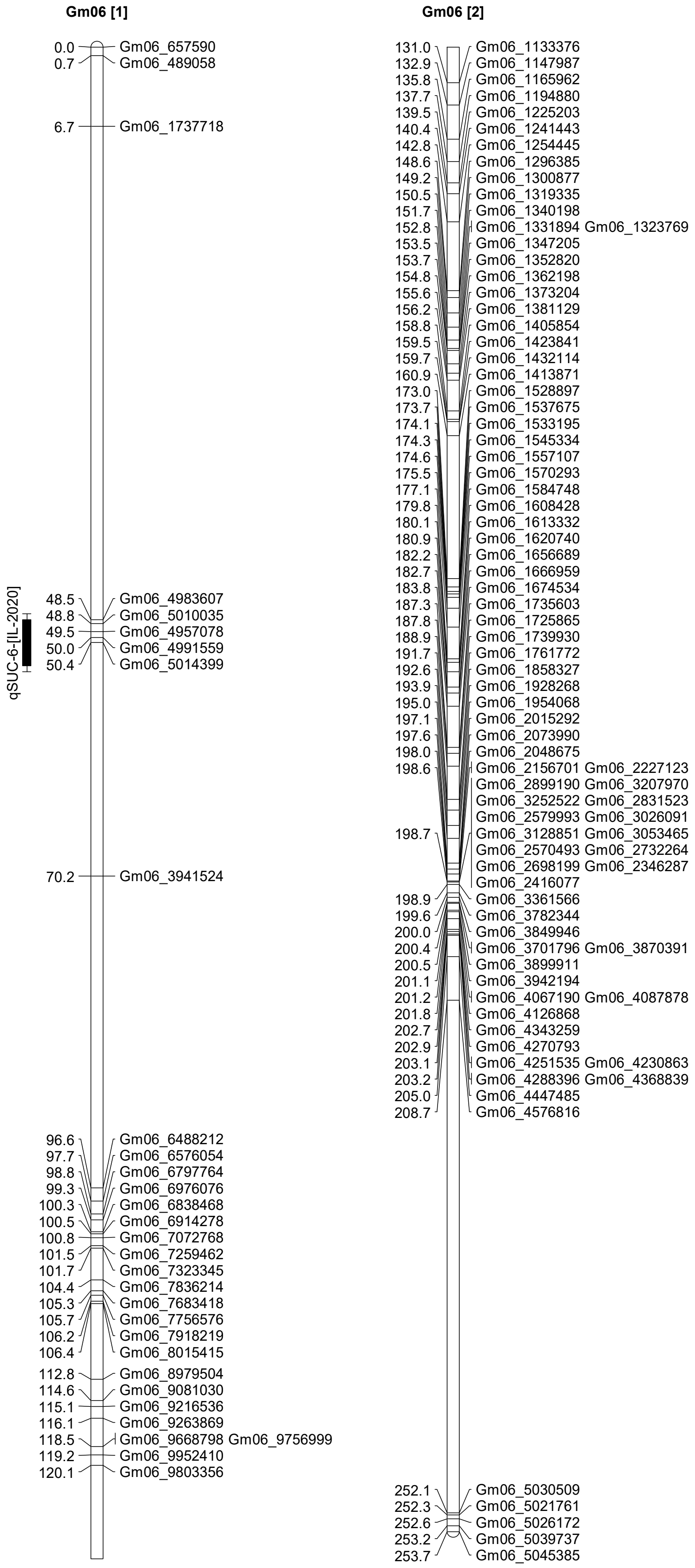
Gm06 [1]
0.0 Gm06_657590
0.7 Gm06_489058
6.7 Gm06_1737718
48.5 Gm06_4983607
48.8 Gm06_5010035
49.5 Gm06_4957078
50.0 Gm06_4991559
50.4 Gm06_5014399
qSUC-6-[IL-2020]
70.2 Gm06_3941524
96.6 Gm06_6488212
97.7 Gm06_6576054
98.8 Gm06_6797764
99.3 Gm06_6976076
100.3 Gm06_6838468
100.5 Gm06_6914278
100.8 Gm06_7072768
101.5 Gm06_7259462
101.7 Gm06_7323345
104.4 Gm06_7836214
105.3 Gm06_7683418
105.7 Gm06_7756576
106.2 Gm06_7918219
106.4 Gm06_8015415
112.8 Gm06_8979504
114.6 Gm06_9081030
115.1 Gm06_9216536
116.1 Gm06_9263869
118.5 Gm06_9668798 Gm06_9756999
119.2 Gm06_9952410
120.1 Gm06_9803356
Gm06 [2]
131.0 Gm06_1133376
132.9 Gm06_1147987
135.8 Gm06_1165962
137.7 Gm06_1194880
139.5 Gm06_1225203
140.4 Gm06_1241443
142.8 Gm06_1254445
148.6 Gm06_1296385
149.2 Gm06_1300877
150.5 Gm06_1319335
151.7 Gm06_1340198
152.8 Gm06_1331894 Gm06_1323769
153.5 Gm06_1347205
153.7 Gm06_1352820
154.8 Gm06_1362198
155.6 Gm06_1373204
156.2 Gm06_1381129
158.8 Gm06_1405854
159.5 Gm06_1423841
159.7 Gm06_1432114
160.9 Gm06_1413871
173.0 Gm06_1528897
173.7 Gm06_1537675
174.1 Gm06_1533195
174.3 Gm06_1545334
174.6 Gm06_1557107
175.5 Gm06_1570293
177.1 Gm06_1584748
179.8 Gm06_1608428
180.1 Gm06_1613332
180.9 Gm06_1620740
182.2 Gm06_1656689
182.7 Gm06_1666959
183.8 Gm06_1674534
187.3 Gm06_1735603
187.8 Gm06_1725865
188.9 Gm06_1739930
191.7 Gm06_1761772
192.6 Gm06_1858327
193.9 Gm06_1928268
195.0 Gm06_1954068
197.1 Gm06_2015292
197.6 Gm06_2073990
198.0 Gm06_2048675
198.6 Gm06_2156701 Gm06_2227123
Gm06_2899190 Gm06_3207970
Gm06_3252522 Gm06_2831523
Gm06_2579993 Gm06_3026091
198.7 Gm06_3128851 Gm06_3053465
Gm06_2570493 Gm06_2732264
Gm06_2698199 Gm06_2346287
Gm06_2416077
198.9 Gm06_3361566
199.6 Gm06_3782344
200.0 Gm06_3849946
200.4 Gm06_3701796 Gm06_3870391
200.5 Gm06_3899911
201.1 Gm06_3942194
201.2 Gm06_4067190 Gm06_4087878
201.8 Gm06_4126868
202.7 Gm06_4343259
202.9 Gm06_4270793
203.1 Gm06_4251535 Gm06_4230863
203.2 Gm06_4288396 Gm06_4368839
205.0 Gm06_4447485
208.7 Gm06_4576816
252.1 Gm06_5030509
252.3 Gm06_5021761
252.6 Gm06_5026172
253.2 Gm06_5039737
253.7 Gm06_5045385

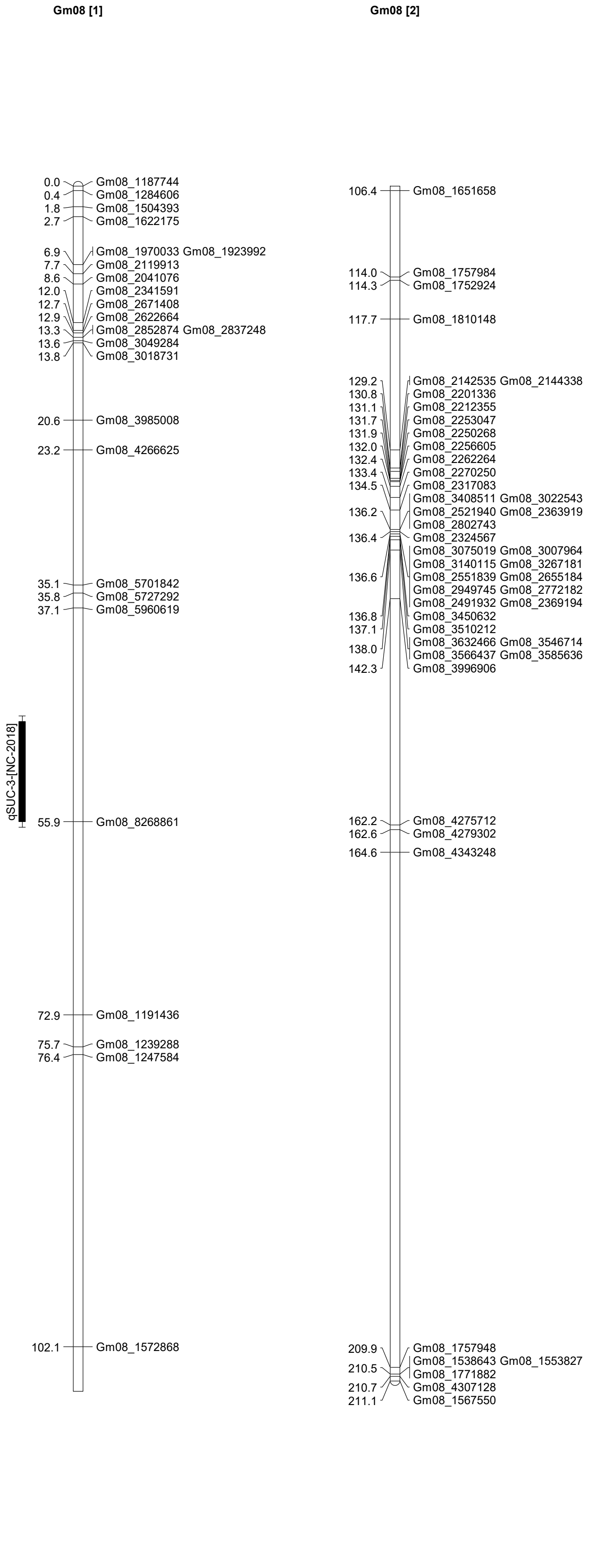
Gm08 [1]
Gm08 [2]
0.0 Gm08_1187744
0.4 Gm08_1284606
1.8 Gm08_1504393
2.7 Gm08_1622175
6.9 Gm08_1970033 Gm08_1923992
7.7 Gm08_2119913
8.6 Gm08_2041076
12.0 Gm08_2341591
12.7 Gm08_2671408
12.9 Gm08_2622664
13.3 Gm08_2852874 Gm08_2837248
13.6 Gm08_3049284
13.8 Gm08_3018731
20.6 Gm08_3985008
23.2 Gm08_4266625
35.1 Gm08_5701842
35.8 Gm08_5727292
37.1 Gm08_5960619
qSUC-3-[NC-2018]
55.9 Gm08_8268861
72.9 Gm08_1191436
75.7 Gm08_1239288
76.4 Gm08_1247584
102.1 Gm08_1572868
106.4 Gm08_1651658
114.0 Gm08_1757984
114.3 Gm08_1752924
117.7 Gm08_1810148
129.2 Gm08_2142535 Gm08_2144338
130.8 Gm08_2201336
131.1 Gm08_2212355
131.7 Gm08_2253047
131.9 Gm08_2250268
132.0 Gm08_2256605
132.4 Gm08_2262264
133.4 Gm08_2270250
134.5 Gm08_2317083
Gm08_3408511 Gm08_3022543
136.2 Gm08_2521940 Gm08_2363919
Gm08_2802743
136.4 Gm08_2324567
Gm08_3075019 Gm08_3007964
Gm08_3140115 Gm08_3267181
136.6 Gm08_2551839 Gm08_2655184
Gm08_2949745 Gm08_2772182
Gm08_2491932 Gm08_2369194
136.8 Gm08_3450632
137.1 Gm08_3510212
138.0 Gm08_3632466 Gm08_3546714
Gm08_3566437 Gm08_3585636
142.3 Gm08_3996906
162.2 Gm08_4275712
162.6 Gm08_4279302
164.6 Gm08_4343248
209.9 Gm08_1757948
Gm08_1538643 Gm08_1553827
210.5 Gm08_1771882
210.7 Gm08_4307128
211.1 Gm08_1567550

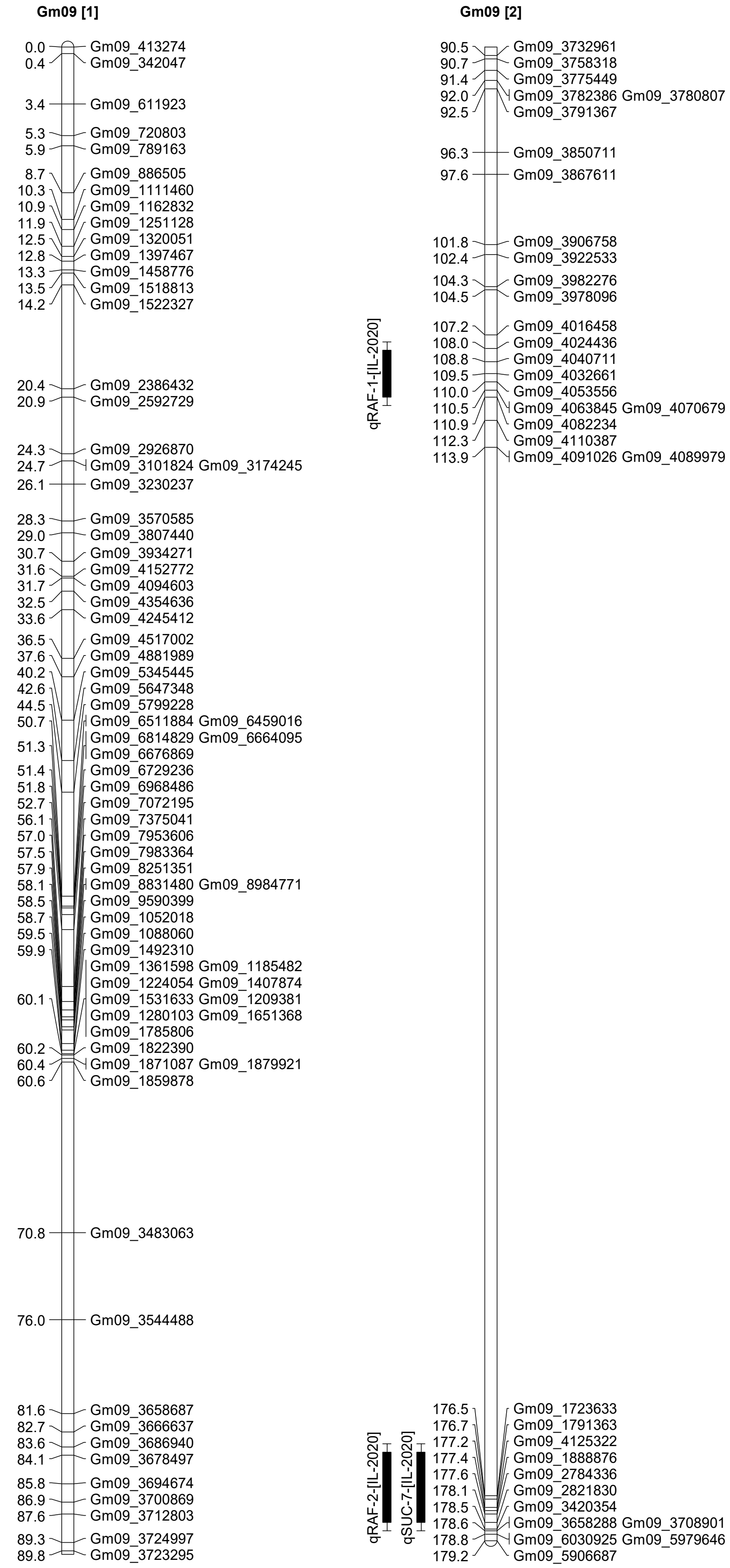
Gm09 [1]
0.0 Gm09_413274
0.4 Gm09_342047
3.4 Gm09_611923
5.3 Gm09_720803
5.9 Gm09_789163
8.7 Gm09_886505
10.3 Gm09_1111460
10.9 Gm09_1162832
11.9 Gm09_1251128
12.5 Gm09_1320051
12.8 Gm09_1397467
13.3 Gm09_1458776
13.5 Gm09_1518813
14.2 Gm09_1522327
20.4 Gm09_2386432
20.9 Gm09_2592729
24.3 Gm09_2926870
24.7 Gm09_3101824 Gm09_3174245
26.1 Gm09_3230237
28.3 Gm09_3570585
29.0 Gm09_3807440
30.7 Gm09_3934271
31.6 Gm09_4152772
31.7 Gm09_4094603
32.5 Gm09_4354636
33.6 Gm09_4245412
36.5 Gm09_4517002
37.6 Gm09_4881989
40.2 Gm09_5345445
42.6 Gm09_5647348
44.5 Gm09_5799228
50.7 Gm09_6511884 Gm09_6459016
Gm09_6814829 Gm09_6664095
51.3 Gm09_6676869
51.4 Gm09_6729236
51.8 Gm09_6968486
52.7 Gm09_7072195
56.1 Gm09_7375041
57.0 Gm09_7953606
57.5 Gm09_7983364
57.9 Gm09_8251351
58.1 Gm09_8831480 Gm09_8984771
58.5 Gm09_9590399
58.7 Gm09_1052018
59.5 Gm09_1088060
59.9 Gm09_1492310
Gm09_1361598 Gm09_1185482
Gm09_1224054 Gm09_1407874
60.1 Gm09_1531633 Gm09_1209381
Gm09_1280103 Gm09_1651368
Gm09_1785806
60.2 Gm09_1822390
60.4 Gm09_1871087 Gm09_1879921
60.6 Gm09_1859878
70.8 Gm09_3483063
76.0 Gm09_3544488
81.6 Gm09_3658687
82.7 Gm09_3666637
83.6 Gm09_3686940
84.1 Gm09_3678497
85.8 Gm09_3694674
86.9 Gm09_3700869
87.6 Gm09_3712803
89.3 Gm09_3724997
89.8 Gm09_3723295
Gm09 [2]
90.5 Gm09_3732961
90.7 Gm09_3758318
91.4 Gm09_3775449
92.0 Gm09_3782386 Gm09_3780807
92.5 Gm09_3791367
96.3 Gm09_3850711
97.6 Gm09_3867611
101.8 Gm09_3906758
102.4 Gm09_3922533
104.3 Gm09_3982276
104.5 Gm09_3978096
107.2 Gm09_4016458
108.0 Gm09_4024436
108.8 Gm09_4040711
109.5 Gm09_4032661
110.0 Gm09_4053556
110.5 Gm09_4063845 Gm09_4070679
110.9 Gm09_4082234
112.3 Gm09_4110387
113.9 Gm09_4091026 Gm09_4089979
qRAF-1-[IL-2020]
176.5 Gm09_1723633
176.7 Gm09_1791363
177.2 Gm09_4125322
177.4 Gm09_1888876
177.6 Gm09_2784336
178.1 Gm09_2821830
178.5 Gm09_3420354
178.6 Gm09_3658288 Gm09_3708901
178.8 Gm09_6030925 Gm09_5979646
179.2 Gm09_5906687
qRAF-2-[IL-2020]
qSUC-7-[IL-2020]

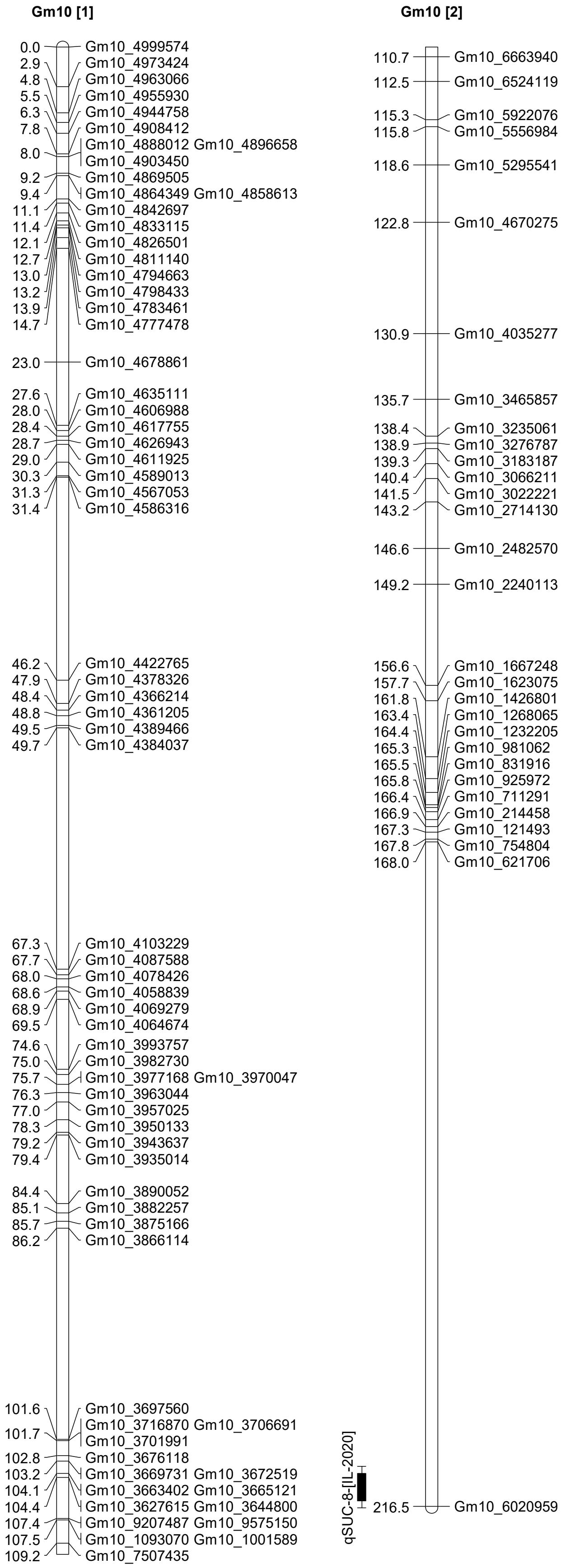
Gm10 [1]
0.0 Gm10_4999574
2.9 Gm10_4973424
4.8 Gm10_4963066
5.5 Gm10_4955930
6.3 Gm10_4944758
7.8 Gm10_4908412
8.0 Gm10_4888012 Gm10_4896658 Gm10_4903450
9.2 Gm10_4869505
9.4 Gm10_4864349 Gm10_4858613
11.1 Gm10_4842697
11.4 Gm10_4833115
12.1 Gm10_4826501
12.7 Gm10_4811140
13.0 Gm10_4794663
13.2 Gm10_4798433
13.9 Gm10_4783461
14.7 Gm10_4777478
23.0 Gm10_4678861
27.6 Gm10_4635111
28.0 Gm10_4606988
28.4 Gm10_4617755
28.7 Gm10_4626943
29.0 Gm10_4611925
30.3 Gm10_4589013
31.3 Gm10_4567053
31.4 Gm10_4586316
46.2 Gm10_4422765
47.9 Gm10_4378326
48.4 Gm10_4366214
48.8 Gm10_4361205
49.5 Gm10_4389466
49.7 Gm10_4384037
67.3 Gm10_4103229
67.7 Gm10_4087588
68.0 Gm10_4078426
68.6 Gm10_4058839
68.9 Gm10_4069279
69.5 Gm10_4064674
74.6 Gm10_3993757
75.0 Gm10_3982730
75.7 Gm10_3977168 Gm10_3970047
76.3 Gm10_3963044
77.0 Gm10_3957025
78.3 Gm10_3950133
79.2 Gm10_3943637
79.4 Gm10_3935014
84.4 Gm10_3890052
85.1 Gm10_3882257
85.7 Gm10_3875166
86.2 Gm10_3866114
101.6 Gm10_3697560
101.7 Gm10_3716870 Gm10_3706691 Gm10_3701991
102.8 Gm10_3676118
103.2 Gm10_3669731 Gm10_3672519
104.1 Gm10_3663402 Gm10_3665121
104.4 Gm10_3627615 Gm10_3644800
107.4 Gm10_9207487 Gm10_9575150
107.5 Gm10_1093070 Gm10_1001589
109.2 Gm10_7507435
Gm10 [2]
110.7 Gm10_6663940
112.5 Gm10_6524119
115.3 Gm10_5922076
115.8 Gm10_5556984
118.6 Gm10_5295541
122.8 Gm10_4670275
130.9 Gm10_4035277
135.7 Gm10_3465857
138.4 Gm10_3235061
138.9 Gm10_3276787
139.3 Gm10_3183187
140.4 Gm10_3066211
141.5 Gm10_3022221
143.2 Gm10_2714130
146.6 Gm10_2482570
149.2 Gm10_2240113
156.6 Gm10_1667248
157.7 Gm10_1623075
161.8 Gm10_1426801
163.4 Gm10_1268065
164.4 Gm10_1232205
165.3 Gm10_981062
165.5 Gm10_831916
165.8 Gm10_925972
166.4 Gm10_711291
166.9 Gm10_214458
167.3 Gm10_121493
167.8 Gm10_754804
168.0 Gm10_621706
216.5 Gm10_6020959
qSUC-8-[IL-2020]

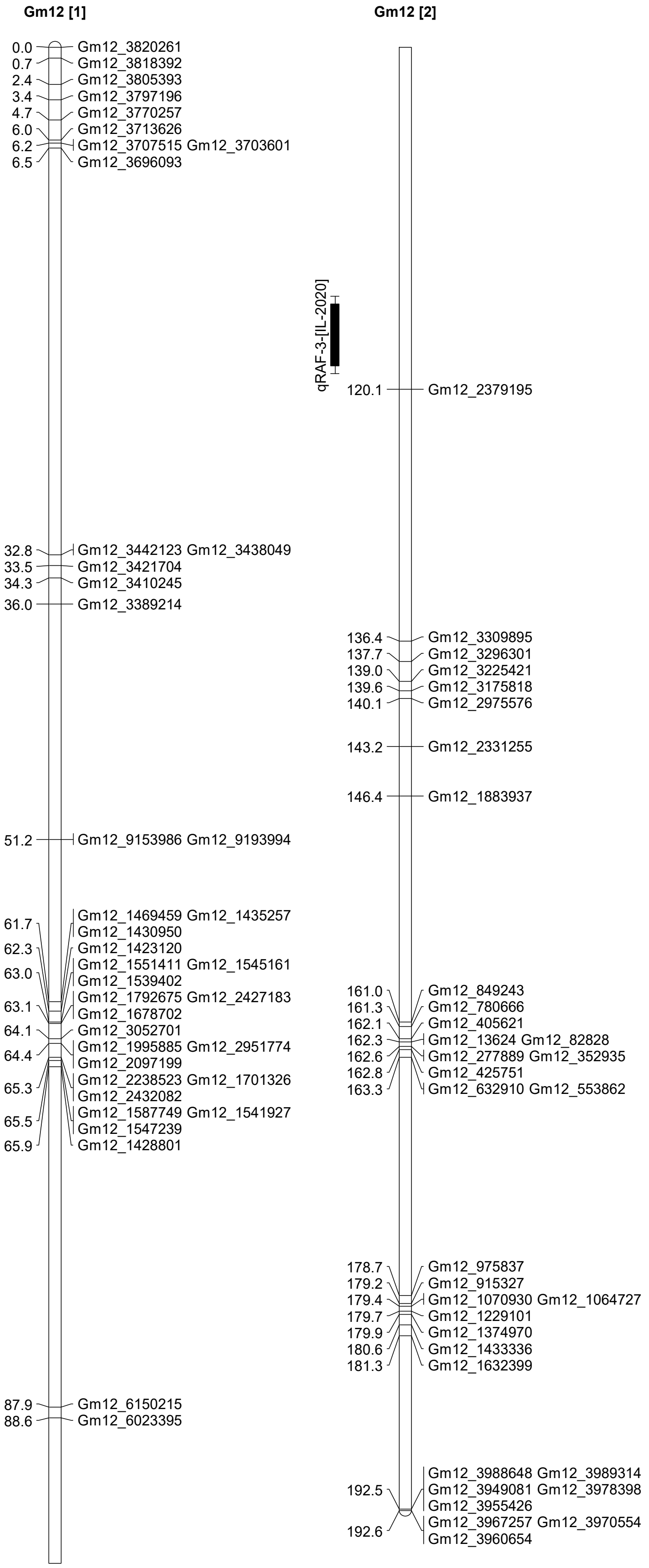
Gm12 [1]
Gm12 [2]
0.0 Gm12_3820261
0.7 Gm12_3818392
2.4 Gm12_3805393
3.4 Gm12_3797196
4.7 Gm12_3770257
6.0 Gm12_3713626
6.2 Gm12_3707515 Gm12_3703601
6.5 Gm12_3696093
32.8 Gm12_3442123 Gm12_3438049
33.5 Gm12_3421704
34.3 Gm12_3410245
36.0 Gm12_3389214
51.2 Gm12_9153986 Gm12_9193994
61.7 Gm12_1469459 Gm12_1435257
Gm12_1430950
62.3 Gm12_1423120
63.0 Gm12_1551411 Gm12_1545161
Gm12_1539402
63.1 Gm12_1792675 Gm12_2427183
Gm12_1678702
64.1 Gm12_3052701
64.4 Gm12_1995885 Gm12_2951774
Gm12_2097199
65.3 Gm12_2238523 Gm12_1701326
Gm12_2432082
65.5 Gm12_1587749 Gm12_1541927
Gm12_1547239
65.9 Gm12_1428801
87.9 Gm12_6150215
88.6 Gm12_6023395
qRAF-3-[IL-2020]
120.1 Gm12_2379195
136.4 Gm12_3309895
137.7 Gm12_3296301
139.0 Gm12_3225421
139.6 Gm12_3175818
140.1 Gm12_2975576
143.2 Gm12_2331255
146.4 Gm12_1883937
161.0 Gm12_849243
161.3 Gm12_780666
162.1 Gm12_405621
162.3 Gm12_13624 Gm12_82828
162.6 Gm12_277889 Gm12_352935
162.8 Gm12_425751
163.3 Gm12_632910 Gm12_553862
178.7 Gm12_975837
179.2 Gm12_915327
179.4 Gm12_1070930 Gm12_1064727
179.7 Gm12_1229101
179.9 Gm12_1374970
180.6 Gm12_1433336
181.3 Gm12_1632399
Gm12_3988648 Gm12_3989314
192.5 Gm12_3949081 Gm12_3978398
Gm12_3955426
Gm12_3967257 Gm12_3970554
192.6 Gm12_3960654

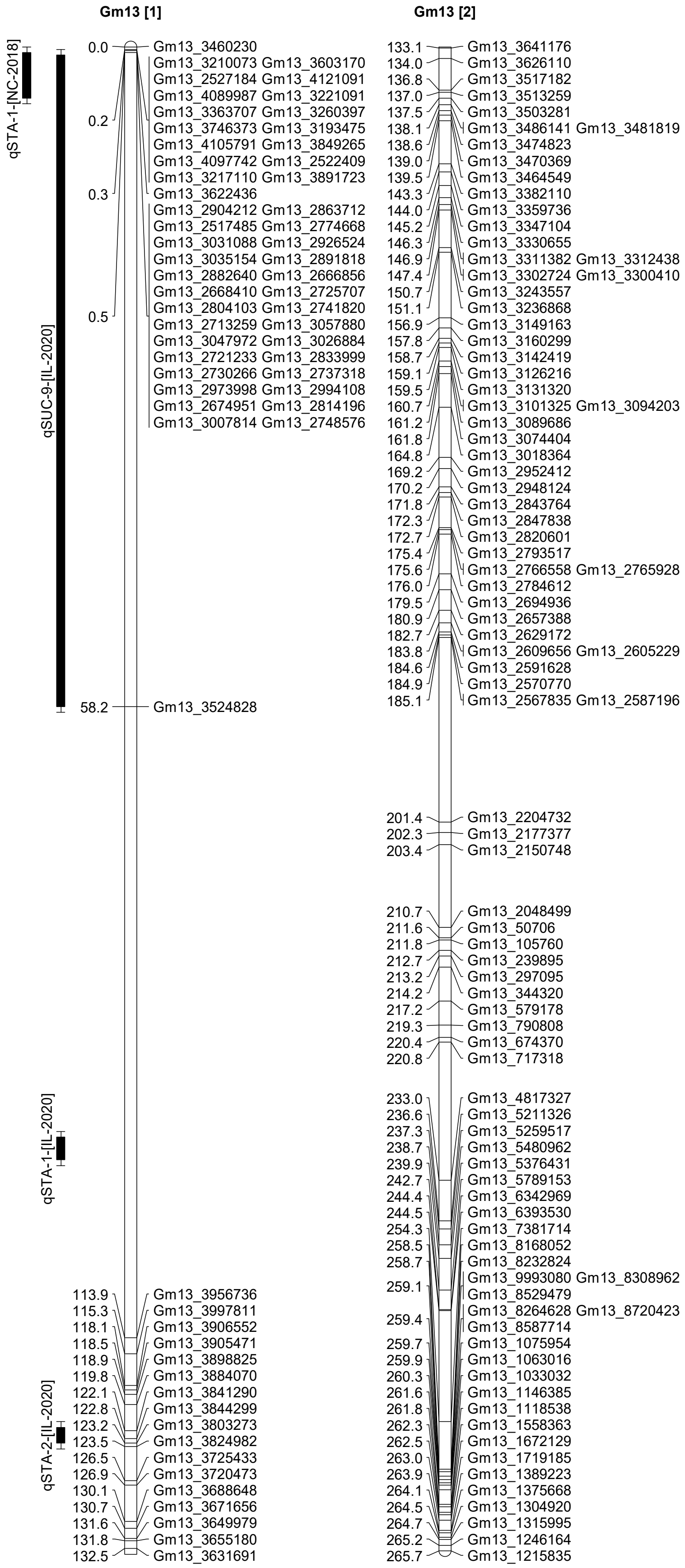
Gm13 [1]
Gm13 [2]
qSTA-1-[NC-2018]
qSUC-9-[IL-2020]
qSTA-1-[IL-2020]
qSTA-2-[IL-2020]
0.0 Gm13_3460230
Gm13_3210073 Gm13_3603170
Gm13_2527184 Gm13_4121091
Gm13_4089987 Gm13_3221091
Gm13_3363707 Gm13_3260397
0.2 Gm13_3746373 Gm13_3193475
Gm13_4105791 Gm13_3849265
Gm13_4097742 Gm13_2522409
Gm13_3217110 Gm13_3891723
0.3 Gm13_3622436
Gm13_2904212 Gm13_2863712
Gm13_2517485 Gm13_2774668
Gm13_3031088 Gm13_2926524
Gm13_3035154 Gm13_2891818
Gm13_2882640 Gm13_2666856
Gm13_2668410 Gm13_2725707
Gm13_2804103 Gm13_2741820
0.5 Gm13_2713259 Gm13_3057880
Gm13_3047972 Gm13_3026884
Gm13_2721233 Gm13_2833999
Gm13_2730266 Gm13_2737318
Gm13_2973998 Gm13_2994108
Gm13_2674951 Gm13_2814196
Gm13_3007814 Gm13_2748576
58.2 Gm13_3524828
113.9 Gm13_3956736
115.3 Gm13_3997811
118.1 Gm13_3906552
118.5 Gm13_3905471
118.9 Gm13_3898825
119.8 Gm13_3884070
122.1 Gm13_3841290
122.8 Gm13_3844299
123.2 Gm13_3803273
123.5 Gm13_3824982
126.5 Gm13_3725433
126.9 Gm13_3720473
130.1 Gm13_3688648
130.7 Gm13_3671656
131.6 Gm13_3649979
131.8 Gm13_3655180
132.5 Gm13_3631691
133.1 Gm13_3641176
134.0 Gm13_3626110
136.8 Gm13_3517182
137.0 Gm13_3513259
137.5 Gm13_3503281
138.1 Gm13_3486141 Gm13_3481819
138.6 Gm13_3474823
139.0 Gm13_3470369
139.5 Gm13_3464549
143.3 Gm13_3382110
144.0 Gm13_3359736
145.2 Gm13_3347104
146.3 Gm13_3330655
146.9 Gm13_3311382 Gm13_3312438
147.4 Gm13_3302724 Gm13_3300410
150.7 Gm13_3243557
151.1 Gm13_3236868
156.9 Gm13_3149163
157.8 Gm13_3160299
158.7 Gm13_3142419
159.1 Gm13_3126216
159.5 Gm13_3131320
160.7 Gm13_3101325 Gm13_3094203
161.2 Gm13_3089686
161.8 Gm13_3074404
164.8 Gm13_3018364
169.2 Gm13_2952412
170.2 Gm13_2948124
171.8 Gm13_2843764
172.3 Gm13_2847838
172.7 Gm13_2820601
175.4 Gm13_2793517
175.6 Gm13_2766558 Gm13_2765928
176.0 Gm13_2784612
179.5 Gm13_2694936
180.9 Gm13_2657388
182.7 Gm13_2629172
183.8 Gm13_2609656 Gm13_2605229
184.6 Gm13_2591628
184.9 Gm13_2570770
185.1 Gm13_2567835 Gm13_2587196
201.4 Gm13_2204732
202.3 Gm13_2177377
203.4 Gm13_2150748
210.7 Gm13_2048499
211.6 Gm13_50706
211.8 Gm13_105760
212.7 Gm13_239895
213.2 Gm13_297095
214.2 Gm13_344320
217.2 Gm13_579178
219.3 Gm13_790808
220.4 Gm13_674370
220.8 Gm13_717318
233.0 Gm13_4817327
236.6 Gm13_5211326
237.3 Gm13_5259517
238.7 Gm13_5480962
239.9 Gm13_5376431
242.7 Gm13_5789153
244.4 Gm13_6342969
244.5 Gm13_6393530
254.3 Gm13_7381714
258.5 Gm13_8168052
258.7 Gm13_8232824
259.1 Gm13_9993080 Gm13_8308962
Gm13_8529479
259.4 Gm13_8264628 Gm13_8720423
Gm13_8587714
259.7 Gm13_1075954
259.9 Gm13_1063016
260.3 Gm13_1033032
261.6 Gm13_1146385
261.8 Gm13_1118538
262.3 Gm13_1558363
262.5 Gm13_1672129
263.0 Gm13_1719185
263.9 Gm13_1389223
264.1 Gm13_1375668
264.5 Gm13_1304920
264.7 Gm13_1315995
265.2 Gm13_1246164
265.7 Gm13_1215835

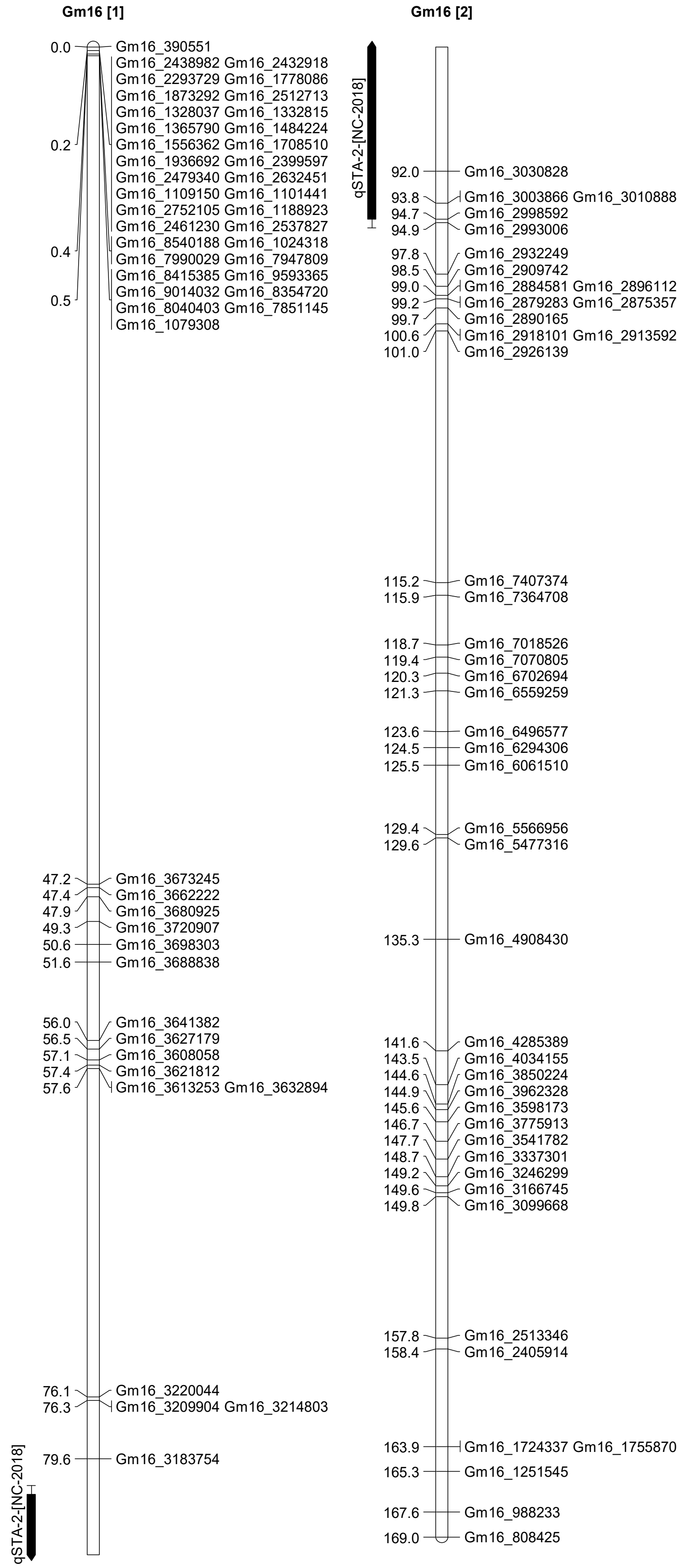

Gm16 [1]
Gm16 [2]
qSTA-2-[NC-2018]
qSTA-2-[NC-2018]
0.0 Gm16_390551
Gm16_2438982 Gm16_2432918
Gm16_2293729 Gm16_1778086
Gm16_1873292 Gm16_2512713
Gm16_1328037 Gm16_1332815
Gm16_1365790 Gm16_1484224
0.2 Gm16_1556362 Gm16_1708510
Gm16_1936692 Gm16_2399597
Gm16_2479340 Gm16_2632451
Gm16_1109150 Gm16_1101441
Gm16_2752105 Gm16_1188923
Gm16_2461230 Gm16_2537827
0.4 Gm16_8540188 Gm16_1024318
Gm16_7990029 Gm16_7947809
Gm16_8415385 Gm16_9593365
0.5 Gm16_9014032 Gm16_8354720
Gm16_8040403 Gm16_7851145
Gm16_1079308
47.2 Gm16_3673245
47.4 Gm16_3662222
47.9 Gm16_3680925
49.3 Gm16_3720907
50.6 Gm16_3698303
51.6 Gm16_3688838
56.0 Gm16_3641382
56.5 Gm16_3627179
57.1 Gm16_3608058
57.4 Gm16_3621812
57.6 Gm16_3613253 Gm16_3632894
76.1 Gm16_3220044
76.3 Gm16_3209904 Gm16_3214803
79.6 Gm16_3183754
92.0 Gm16_3030828
93.8 Gm16_3003866 Gm16_3010888
94.7 Gm16_2998592
94.9 Gm16_2993006
97.8 Gm16_2932249
98.5 Gm16_2909742
99.0 Gm16_2884581 Gm16_2896112
99.2 Gm16_2879283 Gm16_2875357
99.7 Gm16_2890165
100.6 Gm16_2918101 Gm16_2913592
101.0 Gm16_2926139
115.2 Gm16_7407374
115.9 Gm16_7364708
118.7 Gm16_7018526
119.4 Gm16_7070805
120.3 Gm16_6702694
121.3 Gm16_6559259
123.6 Gm16_6496577
124.5 Gm16_6294306
125.5 Gm16_6061510
129.4 Gm16_5566956
129.6 Gm16_5477316
135.3 Gm16_4908430
141.6 Gm16_4285389
143.5 Gm16_4034155
144.6 Gm16_3850224
144.9 Gm16_3962328
145.6 Gm16_3598173
146.7 Gm16_3775913
147.7 Gm16_3541782
148.7 Gm16_3337301
149.2 Gm16_3246299
149.6 Gm16_3166745
149.8 Gm16_3099668
157.8 Gm16_2513346
158.4 Gm16_2405914
163.9 Gm16_1724337 Gm16_1755870
165.3 Gm16_1251545
167.6 Gm16_988233
169.0 Gm16_808425

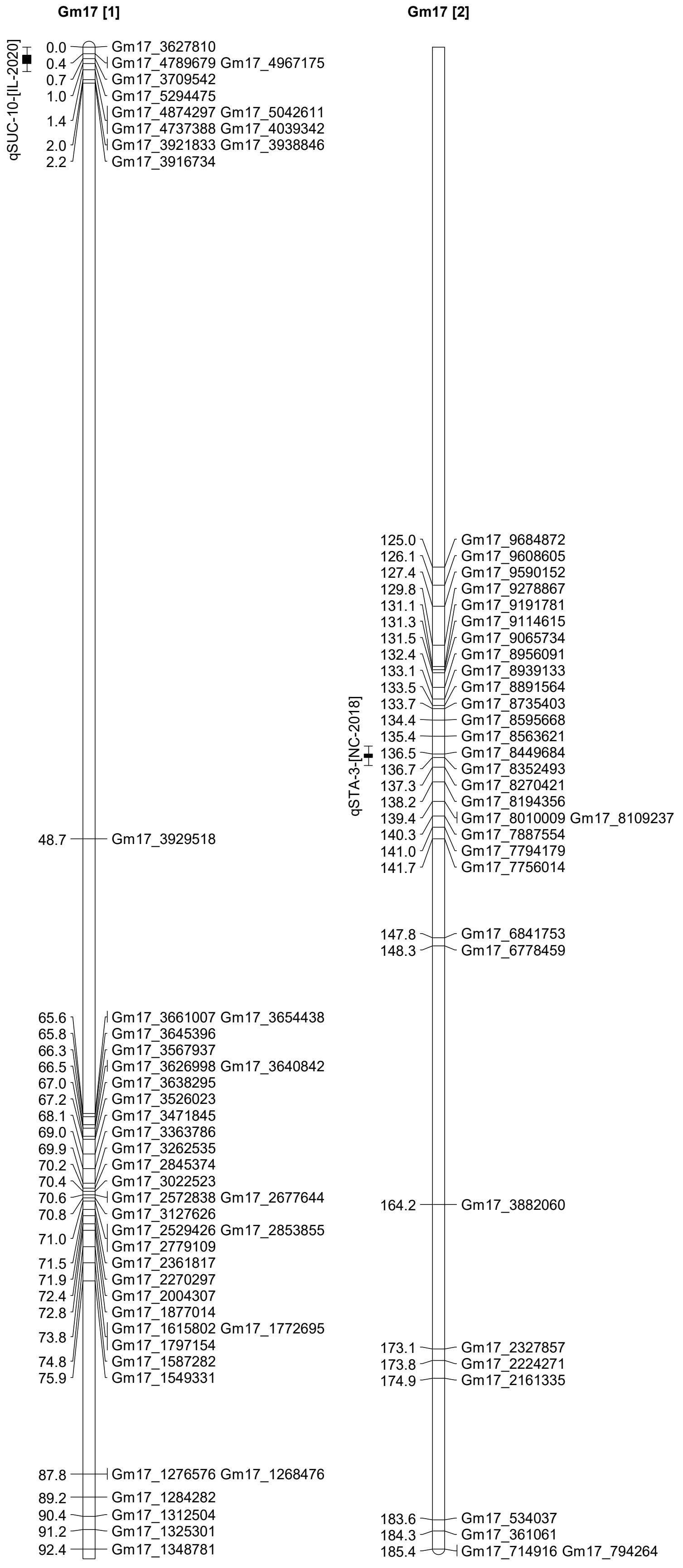
Gm17 [1]
Gm17 [2]
qSUC-10-[IL-2020]
qSTA-3-[NC-2018]
0.0 Gm17_3627810
0.4 Gm17_4789679 Gm17_4967175
0.7 Gm17_3709542
1.0 Gm17_5294475
1.4 Gm17_4874297 Gm17_5042611
Gm17_4737388 Gm17_4039342
2.0 Gm17_3921833 Gm17_3938846
2.2 Gm17_3916734
48.7 Gm17_3929518
65.6 Gm17_3661007 Gm17_3654438
65.8 Gm17_3645396
66.3 Gm17_3567937
66.5 Gm17_3626998 Gm17_3640842
67.0 Gm17_3638295
67.2 Gm17_3526023
68.1 Gm17_3471845
69.0 Gm17_3363786
69.9 Gm17_3262535
70.2 Gm17_2845374
70.4 Gm17_3022523
70.6 Gm17_2572838 Gm17_2677644
70.8 Gm17_3127626
71.0 Gm17_2529426 Gm17_2853855
Gm17_2779109
71.5 Gm17_2361817
71.9 Gm17_2270297
72.4 Gm17_2004307
72.8 Gm17_1877014
73.8 Gm17_1615802 Gm17_1772695
Gm17_1797154
74.8 Gm17_1587282
75.9 Gm17_1549331
87.8 Gm17_1276576 Gm17_1268476
89.2 Gm17_1284282
90.4 Gm17_1312504
91.2 Gm17_1325301
92.4 Gm17_1348781
125.0 Gm17_9684872
126.1 Gm17_9608605
127.4 Gm17_9590152
129.8 Gm17_9278867
131.1 Gm17_9191781
131.3 Gm17_9114615
131.5 Gm17_9065734
132.4 Gm17_8956091
133.1 Gm17_8939133
133.5 Gm17_8891564
133.7 Gm17_8735403
134.4 Gm17_8595668
135.4 Gm17_8563621
136.5 Gm17_8449684
136.7 Gm17_8352493
137.3 Gm17_8270421
138.2 Gm17_8194356
139.4 Gm17_8010009 Gm17_8109237
140.3 Gm17_7887554
141.0 Gm17_7794179
141.7 Gm17_7756014
147.8 Gm17_6841753
148.3 Gm17_6778459
164.2 Gm17_3882060
173.1 Gm17_2327857
173.8 Gm17_2224271
174.9 Gm17_2161335
183.6 Gm17_534037
184.3 Gm17_361061
185.4 Gm17_714916 Gm17_794264

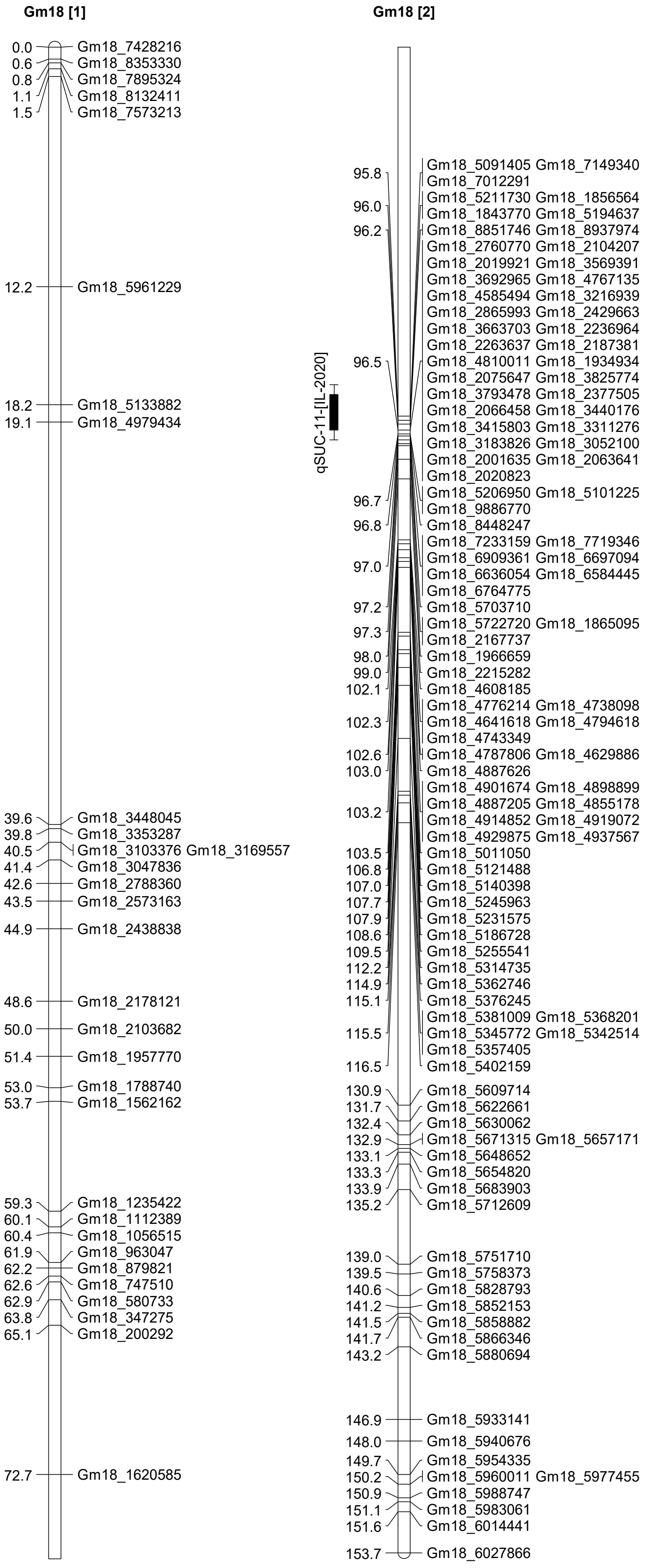
Gm18 [1]
0.0 Gm18_7428216
0.6 Gm18_8353330
0.8 Gm18_7895324
1.1 Gm18_8132411
1.5 Gm18_7573213
12.2 Gm18_5961229
18.2 Gm18_5133882
19.1 Gm18_4979434
39.6 Gm18_3448045
39.8 Gm18_3353287
40.5 Gm18_3103376 Gm18_3169557
41.4 Gm18_3047836
42.6 Gm18_2788360
43.5 Gm18_2573163
44.9 Gm18_2438838
48.6 Gm18_2178121
50.0 Gm18_2103682
51.4 Gm18_1957770
53.0 Gm18_1788740
53.7 Gm18_1562162
59.3 Gm18_1235422
60.1 Gm18_1112389
60.4 Gm18_1056515
61.9 Gm18_963047
62.2 Gm18_879821
62.6 Gm18_747510
62.9 Gm18_580733
63.8 Gm18_347275
65.1 Gm18_200292
72.7 Gm18_1620585
Gm18 [2]
qSUC-11-[IL-2020]
95.8
96.0
96.2
96.5
96.7
96.8
97.0
97.2
97.3
98.0
99.0
102.1
102.3
102.6
103.0
103.2
103.5
106.8
107.0
107.7
107.9
108.6
109.5
112.2
114.9
115.1
115.5
116.5
Gm18_5091405 Gm18_7149340
Gm18_7012291
Gm18_5211730 Gm18_1856564
Gm18_1843770 Gm18_5194637
Gm18_8851746 Gm18_8937974
Gm18_2760770 Gm18_2104207
Gm18_2019921 Gm18_3569391
Gm18_3692965 Gm18_4767135
Gm18_4585494 Gm18_3216939
Gm18_2865993 Gm18_2429663
Gm18_3663703 Gm18_2236964
Gm18_2263637 Gm18_2187381
Gm18_4810011 Gm18_1934934
Gm18_2075647 Gm18_3825774
Gm18_3793478 Gm18_2377505
Gm18_2066458 Gm18_3440176
Gm18_3415803 Gm18_3311276
Gm18_3183826 Gm18_3052100
Gm18_2001635 Gm18_2063641
Gm18_2020823
Gm18_5206950 Gm18_5101225
Gm18_9886770
Gm18_8448247
Gm18_7233159 Gm18_7719346
Gm18_6909361 Gm18_6697094
Gm18_6636054 Gm18_6584445
Gm18_6764775
Gm18_5703710
Gm18_5722720 Gm18_1865095
Gm18_2167737
Gm18_1966659
Gm18_2215282
Gm18_4608185
Gm18_4776214 Gm18_4738098
Gm18_4641618 Gm18_4794618
Gm18_4743349
Gm18_4787806 Gm18_4629886
Gm18_4887626
Gm18_4901674 Gm18_4898899
Gm18_4887205 Gm18_4855178
Gm18_4914852 Gm18_4919072
Gm18_4929875 Gm18_4937567
Gm18_5011050
Gm18_5121488
Gm18_5140398
Gm18_5245963
Gm18_5231575
Gm18_5186728
Gm18_5255541
Gm18_5314735
Gm18_5362746
Gm18_5376245
Gm18_5381009 Gm18_5368201
Gm18_5345772 Gm18_5342514
Gm18_5357405
Gm18_5402159
130.9 Gm18_5609714
131.7 Gm18_5622661
132.4 Gm18_5630062
132.9 Gm18_5671315 Gm18_5657171
133.1 Gm18_5648652
133.3 Gm18_5654820
133.9 Gm18_5683903
135.2 Gm18_5712609
139.0 Gm18_5751710
139.5 Gm18_5758373
140.6 Gm18_5828793
141.2 Gm18_5852153
141.5 Gm18_5858882
141.7 Gm18_5866346
143.2 Gm18_5880694
146.9 Gm18_5933141
148.0 Gm18_5940676
149.7 Gm18_5954335
150.2 Gm18_5960011 Gm18_5977455
150.9 Gm18_5988747
151.1 Gm18_5983061
151.6 Gm18_6014441
153.7 Gm18_6027866

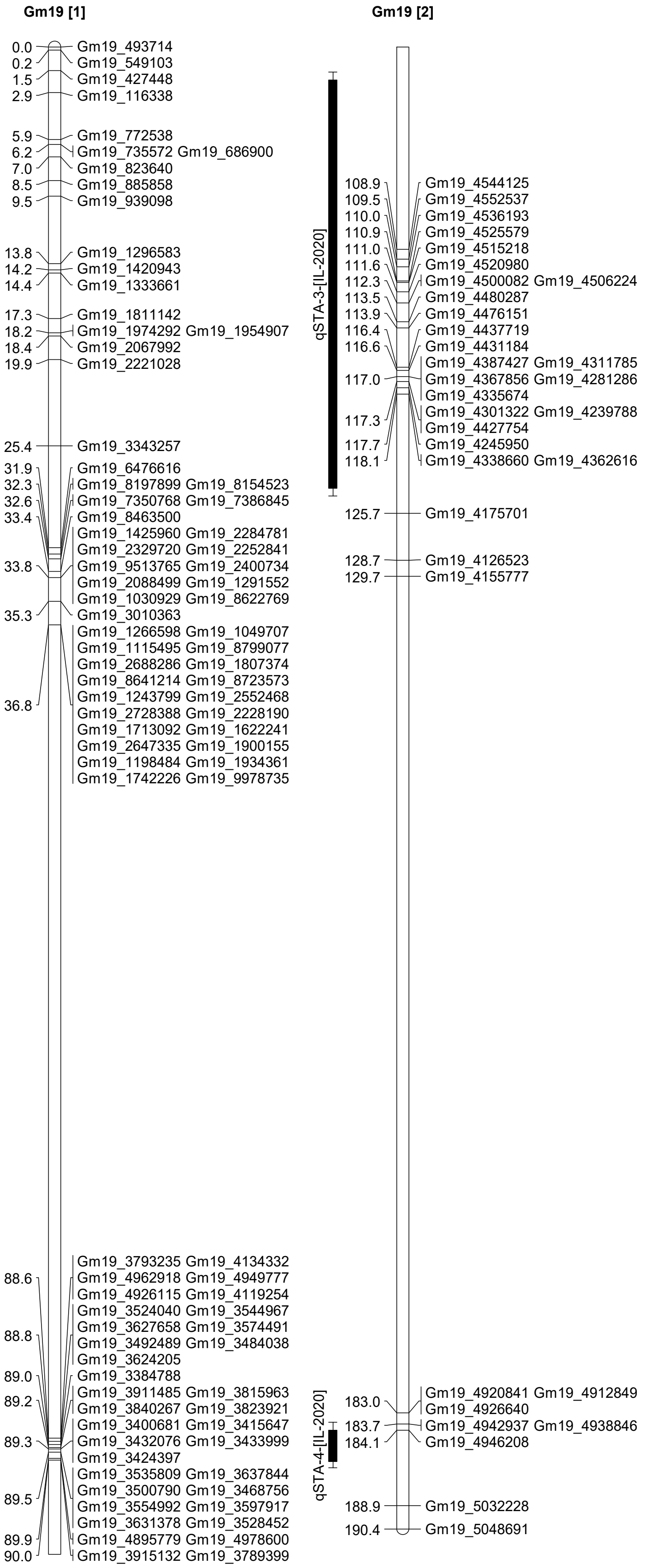
Gm19 [1]
Gm19 [2]
qSTA-3-[IL-2020]
qSTA-4-[IL-2020]

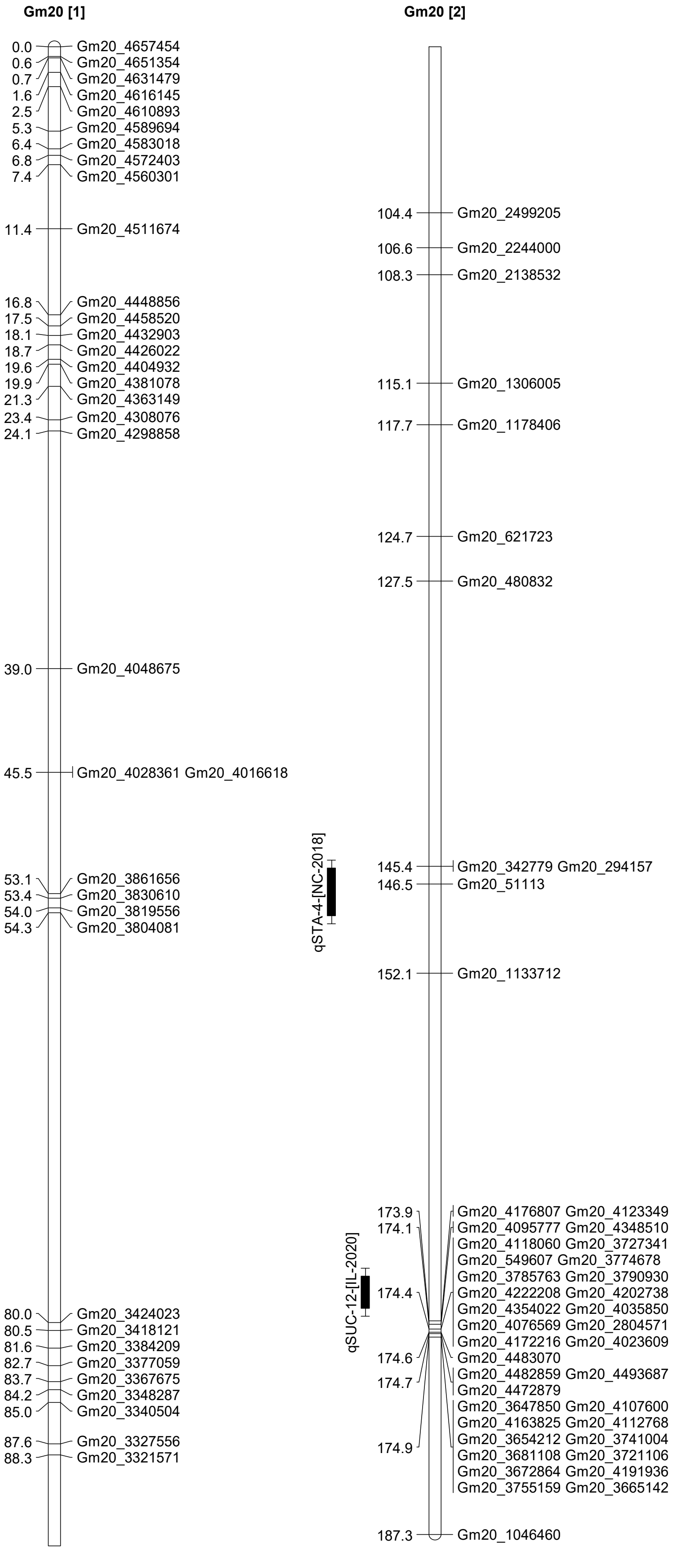

Figure S1. Positions of QTL that control seed sucrose (qSUC), stachyose (qSTA), and raffinose (qRAF) contents on Chrs. 1, 2, 3, 4, 5, 6, 8, 9, 10, 12, 13, 16, 17, 18, 19, and 20. QTL names are followed by a number, location, and year in which they are identified. For example, qSUC-1-(NC-2018).

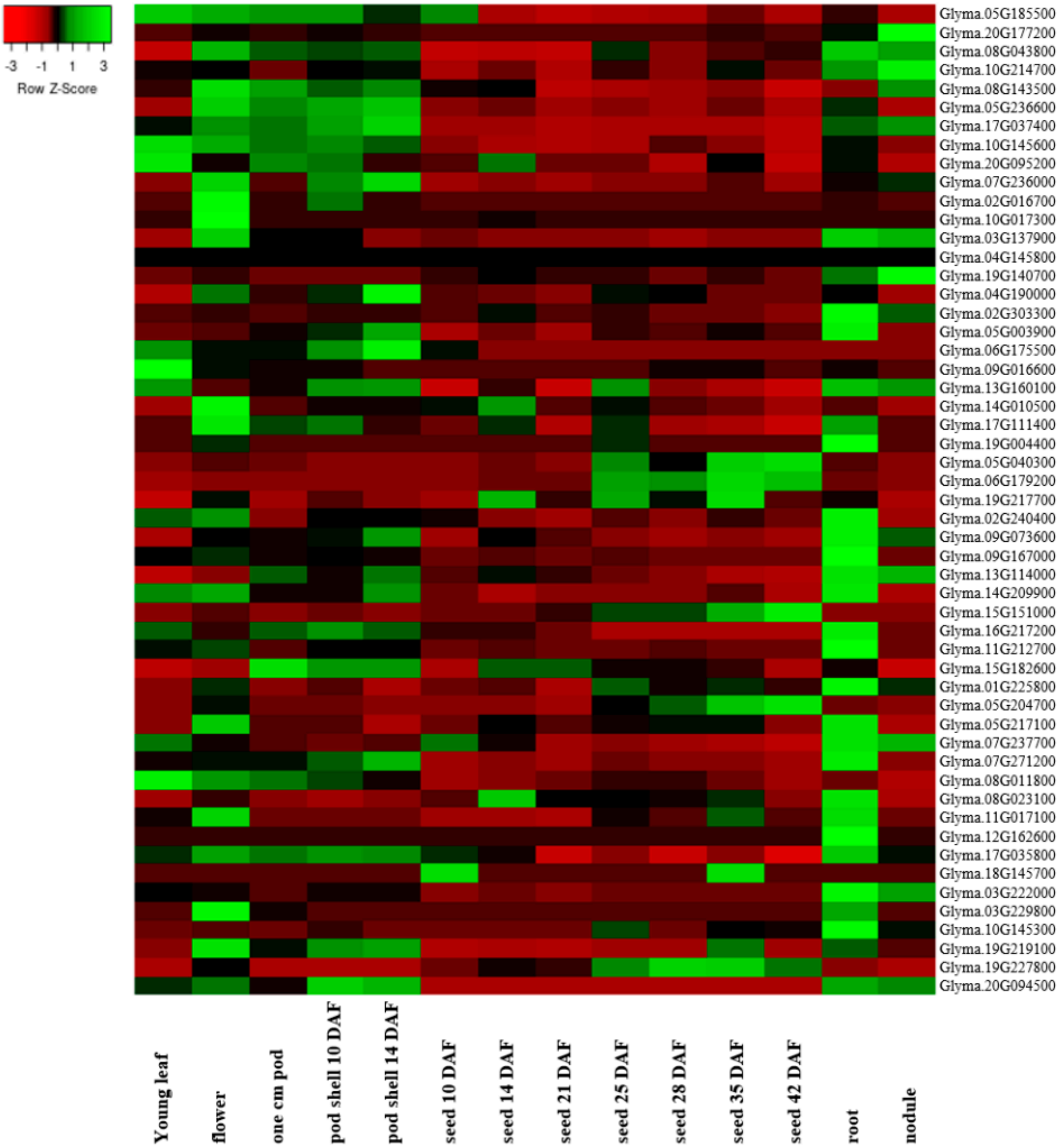

Figure S2. Expression profiles of the sugars (sucrose, raffinose, and stachyose) pathway candidate genes in soybean based on RNAseq data available from RNAsequencing data [20]. RNA-seq data is not available at Soybase for the *Glyma.03G216300*, *Glyma.17G045800*, *Glyma.19G212800*, and *Glyma.18G211700* candidate genes.

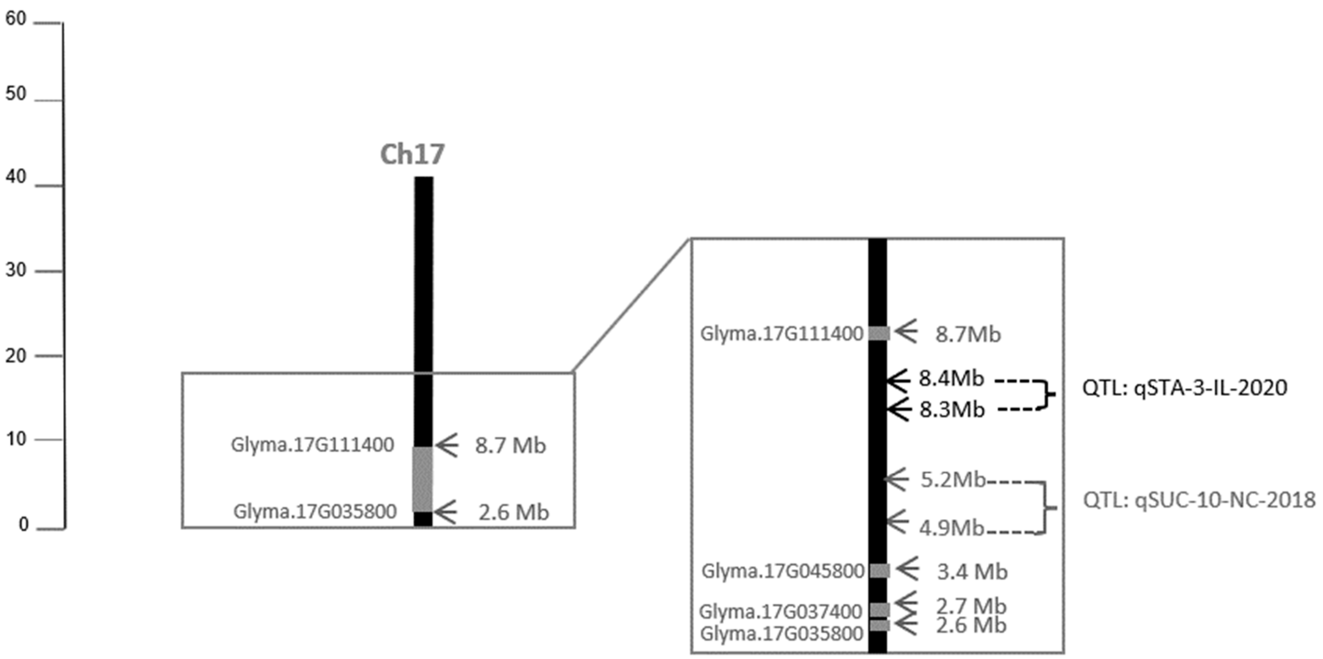

Figure S3. Physical positions corresponding to the *Glyma.17G037400* encoding for an invertase, *Glyma.17G045800* encoding for sucrose synthase, *Glyma.17G111400* encoding for raffinose synthase, and *Glyma.17G035800* encoding for UDP-D-glucose-4-epimerase, and the identified seed sugars QTL identified in this study on chr. 17 are shown.
